# High mobility group box 1 contributes to anti-neutrophil cytoplasmic antibody-induced neutrophils activation through receptor for advanced glycation end products (RAGE) and Toll-like receptor 4

**DOI:** 10.1186/s13075-015-0587-4

**Published:** 2015-03-18

**Authors:** Chen Wang, Huan Wang, Dong-Yuan Chang, Jian Hao, Ming-Hui Zhao, Min Chen

**Affiliations:** Department of Medicine, Renal Division, Peking University First Hospital; Institute of Nephrology, Peking University, Key Laboratory of Renal Disease, Ministry of Health of China, 8, Xishiku Street, Beijing, 100034 China

## Abstract

**Introduction:**

High mobility group box-1 (HMGB1), a typical damage-associated molecular pattern (DAMP) protein, is associated with inflammatory conditions and tissue damage. Our recent study found that circulating HMGB1 levels could reflect the disease activity of antineutrophil cytoplasmic antibody (ANCA)-associated vasculitis (AAV). The current study aimed to investigate whether HMGB1 participated in ANCA-induced neutrophil activation, which is one of the most important pathogenic aspects in the development of AAV.

**Methods:**

The various effects of HMGB1 in ANCA-induced neutrophil activation were measured. Antagonists for relevant receptors and signaling molecules were employed.

**Results:**

ANCA antigens translocation on neutrophils primed with HMGB1 was significantly higher than non-primed neutrophils. The levels of respiratory burst and degranulation increased significantly in HMGB1-primed neutrophils activated with ANCA-positive IgG, as compared with non-primed neutrophils. Furthermore, blocking Toll-like receptor 4 (TLR4) and receptor for advanced glycation end products (RAGE), rather than TLR2, resulted in a significant decrease in HMGB1-induced ANCA antigens translocation, respiratory burst and degranulation. Similar effects were also found when blocking MyD88 and NF-κB.

**Conclusions:**

HMGB1 could prime neutrophils by increasing ANCA antigens translocation, and the primed neutrophils could be further induced by ANCA, resulting in the respiratory burst and degranulation. This process is TLR4- and RAGE-dependent through the MyD88/NF-κB pathway.

**Electronic supplementary material:**

The online version of this article (doi:10.1186/s13075-015-0587-4) contains supplementary material, which is available to authorized users.

## Introduction

Antineutrophil cytoplasmic antibody (ANCA)-associated vasculitis (AAV) consists of granulomatosis with polyangiitis (GPA, previously named Wegener’s granulomatosis), microscopic polyangiitis (MPA) and eosinophilic granulomatosis with polyangiits (EGPA) [1]. The serological markers for the aforementioned primary small vessel vasculitis were ANCAs, which recognize a variety of target antigens, in particular, proteinase 3 (PR3) and myeloperoxidase (MPO).

ANCA-induced neutrophil activation is increasingly being recognized to play an important role in the pathogenesis of AAV. Cytokines or other proinflammatory mediator, such as C5a and tumor necrosis factor-α (TNF-α), could prime neutrophils by inducing more ANCA antigens expression on the surface of neutrophils. Thus, ANCAs could further activate primed neutrophils to undergo a respiratory burst and degranulation, which plays a direct pathogenic role in the development of vasculitis [2-6]. Furthermore, it was demonstrated that in animal studies that ANCA and neutrophils were necessary for the pathogenesis of AAV [7,8].

High mobility group box-1 (HMGB1) exists within the nucleus ubiquitously, playing its nuclear role by stabilizing the structure of nucleosomes and inducing DNA bending [9]. In recent years, a novel role of HMGB1 as a typical damage-associated molecular pattern (DAMP) protein when placed extracellularly has been attracting increasing attention [10]. The signal pathways of HMGB1 involve a number of signaling molecules and receptors, including receptor for advanced glycation end products (RAGE) and Toll-like receptors (TLR) 2 and 4, may participate in HMGB1 signaling [11-13].

In our recent study, we observed circulating HMGB1 levels are closely associated the disease activity of AAV [14]. Therefore, it is reasonable to further investigate whether HMGB1, a proinflammatory mediator, plays a pathogenic role in the development of AAV. It is noticed that HMGB1 has a variety of effects on neutrophils, which are the most important effector cells in the pathogenesis of AAV. Fan *et al.* reported that HMGB1/TLR4 signaling attributed to the activation of neutrophils NADPH oxidase, which further induced neutrophil-mediated inflammation and organ injury after hemorrhage [15]. *In vitro*, HMGB1 played an essential regulatory role in neutrophil recruitment and migration to inflammatory tissues [16]. Therefore, we hypothesized that the HMGB1 participates in ANCA-induced neutrophil activation. Furthermore, in the current study, we also investigated receptors and intracellular signaling pathways involved in HMGB1-primed neutrophils for ANCA-induced activation.

## Materials and methods

### Reagents

Recombinant HMGB1 proteins were purchased from R&D Systems (C23-C45 disulfide C106 thiol form) (Abingdon, UK). The endotoxin level of HMGB1 was below the detection limit (0.125 EU/mL) of the Limulus assay (Sigma-Aldrich, St Louis, MO, USA). Recent studies found the functionality of HMGB1 is affected by the redox state of its three cysteine residues (C23, C45 and C106). The intramolecular disulfide bond between Cys23 and Cys45 as well as the presence of the unpaired Cys106 thiol were critical for HMGB1-induced proinflammatory activity [17,18]. The recombinant HMGB1 used in our study has been adjusted to the above form to ensure proinflammatory activity.

Antibodies blocking the TLR2 and TLR4 were purchased from eBioscience (San Diego, CA, USA). RAGE-Fc was purchased from R&D Systems (Minneapolis, MN, USA). Inhibitor of myeloid differentiation factor 88 (MyD88) was purchased from Imgenex (San Diego, CA, USA), while inhibitor of nuclear factor kappa B (NF-κB) (Bay11–7082) was from Biovision (Mountain View, CA, USA). For flow cytometry analysis, rabbit polyclonal antibody against human PR3 was purchased from Abcam (Cambridge, UK), with addition of irrelevant IgG control antibody and phycoerythrin (PE)-conjugated goat anti-rabbit antibody. Fluorochrome dihydrorhodamine was purchased from Sigma-Aldrich (St Louis, MO, USA). For Western blot analysis, antibodies against MyD88 (D80F5) and NF-κB p65 (Ser536) were purchased from Cell Signaling Technology (Beverly, MA, USA).

### Preparation of IgG

Normal immunoglobulin G (IgG) and ANCA-positive IgG were prepared from plasma of normal volunteers and patients with active PR3-ANCA- or MPO-ANCA-positive primary small vessel vasculitis, using a High-Trap-protein G column on an AKTA-FPLC system (GE Biosciences, South San Francisco, CA, USA). None of these patients had dual positivity of PR3-ANCA and MPO-ANCA. Preparation of IgG was performed according to the methods described previously [19,20]. We obtained written informed consent from all participants involved in our study. The research was in compliance of the Declaration of Helsinki and approved by the clinical research ethics committee of the Peking University First Hospital.

### Neutrophil isolation

Neutrophils were isolated from heparinized venous blood of healthy donors by density gradient centrifugation on PolymorphPrep (Nycomed, Oslo, Norway). Then neutrophils were washed in Hank’s balanced salt solution without Ca^2+^/Mg^2+^ (HBSS−/−; Chemical Reagents, Beijing, China) and suspended in HBSS with Ca^2+^/Mg^2+^ (HBSS+/+; Chemical Reagents, Beijing, China) to a concentration of 1 × 10^6^ cells/ml and used for further analysis.

### Cytotoxicity assay

The effect of various blocking antibodies and inhibitors on cell toxicity was determined by measuring the secretion of lactate dehydrogenase (LDH) using the Cytotoxicity Detection Kit (Roche Diagnostics, Mannheim, Germany) according to manufacturer’s recommendation.

### Membrane expression of PR3 (mPR3) on neutrophils after priming

**Figure 1:**
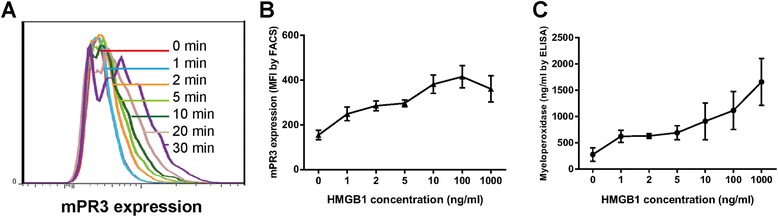
**Dose–response curve for HMGB1 in priming neutrophils and a representative histogram of effects of HMGB1 on translocation of PR3 over time. (A)** was a representative histogram of effects of HMGB1 on translocation of PR3 over time. The levels of mPR3 expression on neutrophils **(B)** and MPO in the supernatant of neutrophils **(C)** was roughly dose-dependent. Bars represent mean ± standard deviation (SD) of repeated measurements of three independent experiments. HMGB1, high mobility group box-1; MPO, myeloperoxidase; PR3, proteinase 3.

**Figure 2:**
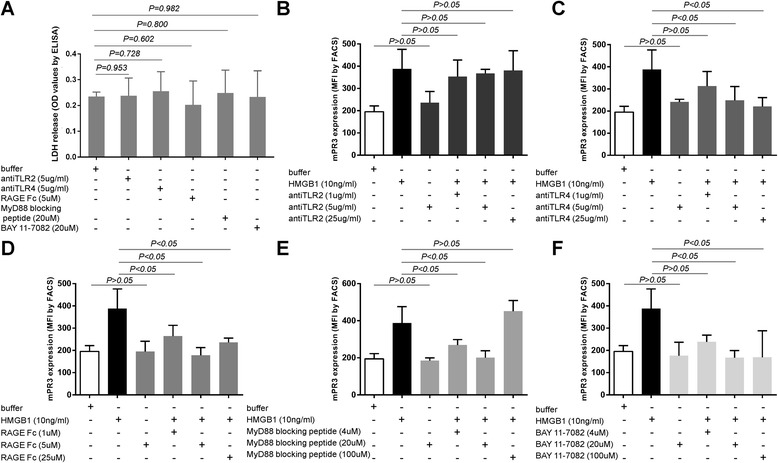
**Cytotoxicity assay of neutrophils with different stimulations and dose–response curves for involved blocking antibodies and inhibitors.** LDH release of neutrophils incubated with anti-TLR2 at 5 μg/ml, anti-TLR4 at 5 μg/ml, RAGE-Fc at 5 μM, MyD88 inhibitor at 20 μM or NF-κB inhibitor were measured. Involved blocking antibodies and inhibitors showed no significantly cytotoxicity **(A)** activating effect **(B-F)** to neutrophils. B-F also showed dose–response curves for involved blocking antibodies and inhibitors. Bars represent mean ± standard deviation (SD) of repeated measurements of three independent experiments. LDH, lactate dehydrogenase; MyD88, myeloid differentiation factor 88; NF-κB, nuclear factor kappa B; RAGE, receptor for advanced glycation end products; TLR, Toll-like receptor.

Flow cytometry was used to evaluate PR3 expression on neutrophils. Neutrophils were incubated with a concentration of HMGB1 at 10 ng/ml, which was comparable to the circulating HMGB1 level in active AAV patients, as demonstrated in our previous study [14] or buffer control, for 30 min at 37°C. The time was set according to the result of our finding that the mPR3 expression increased gradually over time within 30 min. To avoid influence on the activity and survival of cells with longer time, the priming time was set to 30 min (Figure 1A). In order to investigate the role of candidate receptors and signaling molecules in HMGB1-primed neutrophils, neutrophils were first incubated with blocking antibodies and inhibitors (anti-TLR2 at 5 μg/ml; anti-TLR4 at 5 μg/ml; RAGE-Fc at 5 μM; MyD88 inhibitor at 20 μM; NF-κB inhibitor at 20 μM, the above inhibiting concentrations were set according to dose-dependent curve (Figure 2B-F) or buffer control for 1 h on ice. Neutrophils were incubated with 0.5 mg/ml heat-aggregated goat IgG for 15 min to saturate Fcγ receptors. Next, cells were stained with a saturating dose of rabbit polyclonal antibody against human PR3 or with an irrelevant IgG control antibody for 30 min on ice. Neutrophils were then incubated with PE-conjugated goat anti-rabbit antibody. All further steps were performed on ice and washing steps were carried out using HBSS +/+ containing 1% bovine serum albumin (BSA). Prepared neutrophils were analyzed using a FACScan (Becton Dickinson, Heidelberg, Germany) as described previously [21]. Cells allocated like neutrophils were gated in forward/sideward scatter (FSC/SSC) and data were collected from 10,000 cells per sample.

### Detection of MPO in the supernatant of HMGB1-primed neutrophils by ELISA

MPO in the supernatant of HMGB1-primed neutrophils was tested by enzyme-linked immunosorbent assay (ELISA) using a commercial kit (USCNK, Wuhan, China). Cells were incubated with HMGB1 (10 ng/ml) for 30 min at 37°C. Supernatant fluids were collected and used for ELISA analysis. Assay of role of candidate receptors and signaling molecules was described as above. The ELISA procedure of measuring MPO was as described previously [22].

### Measurement of respiratory burst by oxidation of dihydrorhodamine to rhodamine

We assessed the generation of reactive oxygen radicals using dihydrorhodamine, as described previously [23]. In brief, isolated neutrophils were gradually warmed to 37°C and incubated with 0.05 mM dihydrorhodamine for 10 min at 37°C. Sodium azide (NaN_3_) (2 mM) was added in order to prevent intracellular breakdown of H_2_O_2_ by catalase. Then, neutrophils were primed with 10 ng/ml HMGB1 for 30 min at 37°C and incubated with patient-derived ANCA-positive-IgG (300 μg/ml) or normal IgGs (300 μg/ml) for 1 h at 37°C. For assay of role of candidate receptors and signaling molecules, certain groups of neutrophils were pre-incubated with relevant reagents for 1 h on ice before the priming. The reaction was stopped by addition of 1 ml of ice-cold HBSS/1% BSA. Samples were kept on ice and analyzed using a FACScan. Cells allocated like neutrophils were gated in forward/sideward scatter (FSC/SSC) and data were collected from 10,000 cells per sample. The shift of green fluorescence in the FL-1 mode was determined. For each condition, the mean fluorescence intensity (MFI) (representing the amount of generated reactive oxygen radicals) was reported [21,24].

### ANCAs activated HMGB1-primed neutrophils degranulation

Lactoferrin, an iron-binding multifunctional glycoprotein that was an abundant component of the specific granules of neutrophils [25], was considered as a biomarker of neutrophil degranulation [26]. Neutrophils were stimulated with HMGB1 10 ng/ml or buffer for 30 min followed by stimulation with MPO-ANCA-positive IgG or PR3-ANCA-positive IgG, or normal IgGs for 1 h, respectively. For assay of role of candidate receptors and signaling molecules, certain groups of neutrophils were pre-incubated with relevant reagents for 1 h on ice before the priming. Supernatant fluids were collected and used for ELISA analysis. Lactoferrin in the neutrophils supernatant were tested by ELISA using a commercial kit according to the manufacturer’s instruction (USCNK, Wuhan, China).

### Western blot analysis of MyD88 and phospho-NF-κB

MyD88 is the common adaptor for almost all TLRs, and RAGE as well. The receptors associate with MyD88 through their TIR domains, recruiting serine/threonine kinases interleukin-1 receptor-associated kinases (IRAKs) to stimulate the TNF receptor-associated factor 6/IκB kinase (TRAF6/IKK) complex and mitogen-activated protein kinase-kinase (MAPKK), which result in activation of NF-κB [27,28]. Here MyD88 and NF-κB were taken into investigation.

Neutrophils were stimulated with HMGB1 10 ng/ml or buffer for 30 min followed by stimulation with MPO-ANCA-positive IgG or PR3-ANCA-positive IgG, normal IgG or buffer control for 1 h, respectively. Samples were harvested and cell lysates were prepared using the NE-PER Nuclear and Cytoplasmic Extraction Kit (Pierce, Rockford, IL, USA). The concentrations of samples were determined using a BCA protein assay kit (Sigma-Aldrich, Poole, UK). Samples were incubated for 5 min at 95°C in loading buffer and 30 μg of protein per lane were loaded on 10% sodium dodecyl sulfate-polyacrylamide gel, electrophoresed, and blotted onto nitrocellulose membrane by semidry equipment. Membrane was blocked in 5% BSA/0.05% Tween 20/Tris-buffered saline (TBST) 1 h at room temperature. The target bands were detected using specific monoclonal rabbit antibodies against MyD88 and phospho-NF-κB p65 (1:1,000 dilution in 5%BSA/TBST with a final concentration of 1 μg/ml) or mouse antibody against GAPDH (1:500 dilution in 5% BSA/TBST with a final concentration of 1 μg/ml). The nitrocellulose membrane was incubated overnight at 4°C with gentle agitation, followed by three washes with TBST for 10 min each time. The strips were then incubated with peroxidase-conjugated affinity-purified goat anti-mouse IgG or goat anti-rabbit IgG (Sigma-Aldrich, St Louis, MO, USA) diluted at 1:10,000 with TBST/5% BSA for 1 h at room temperature with gentle agitation. Finally, they were revealed on autoradiographic film using ECL Plus Western Blotting Detection System (GE Healthcare, Piscataway, NJ, USA).

### Statistical analysis

Quantitative data were expressed as means ± standard deviation (SD) (for data that were normally distributed) or median and quartiles (for data that were not normally distributed) as appropriated. Differences of quantitative parameters between groups were assessed using one-way analysis of variance (ANOVA) analysis (for data that were normally distributed) or Mann–Whitney *U* test (for data that were not normally distributed) as appropriate. Differences were considered significant when *P* <0.05. Analysis was performed with SPSS statistical software package (version 13.0, SPSS Inc., Chicago, IL, USA).

## Results

### The effect of HMGB1 on neutrophils was dose-dependent

First, neutrophils were incubated with various concentrations of HMGB1 (1, 2, 5, 10, 100 and 1000 ng/ml), and mPR3 expression was determined by flow cytometry. The level of mPR3 expression on neutrophils was roughly dose-dependent (Figure 1B). Then MPO in the supernatant of neutrophils primed by these concentrations of HMGB1 was then tested. The level of MPO in the supernatant of neutrophils was also dose-dependent (Figure 1C).

### HMGB1 increased the expression of mPR3 on neutrophils and the concentration of MPO in the supernatant of neutrophils

**Figure 3:**
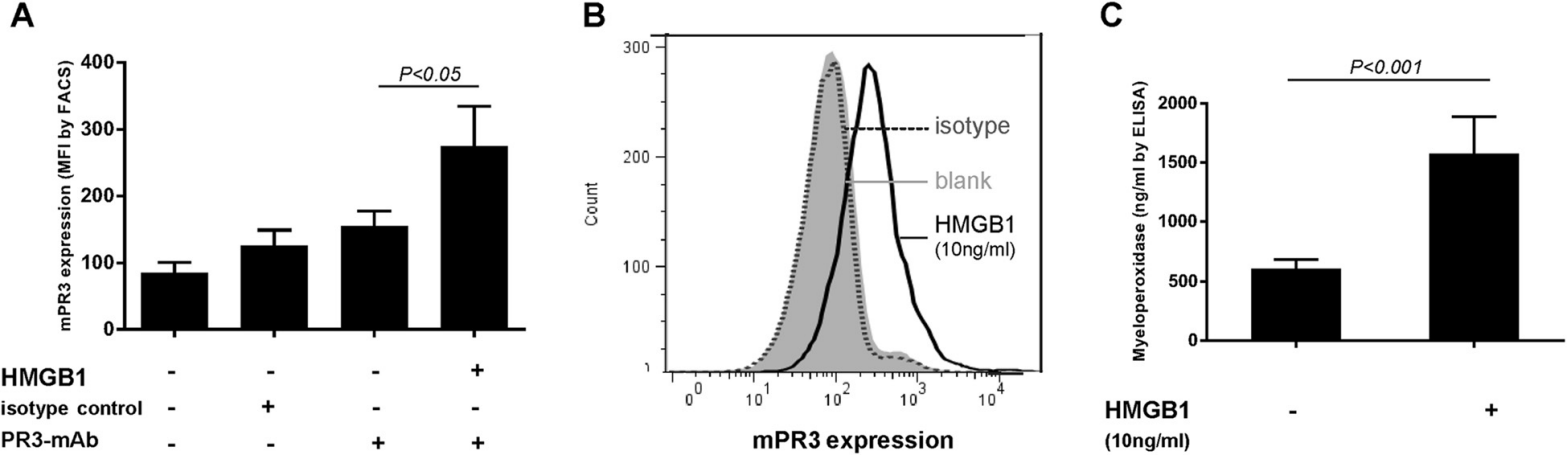
**ANCA antigens translocation enhanced by incubation of HMGB1.** HMGB1 increased expression of mPR3 on neutrophils **(A)** and concentration of MPO in the neutrophils culture supernatant **(C)**. **(B)** was a representative histogram of effects of HMGB1 on translocation of PR3. Bars represent mean ± standard deviation (SD) of repeated measurements on neutrophils of eight independent experiments and donors. ANCA, antineutrophil cytoplasmic antibody; HMGB1, high mobility group box-1; MPO, myeloperoxidase; PR3, proteinase 3.

Expression of mPR3 on neutrophils and the concentration of MPO in the supernatant of HMGB1-primed neutrophils of eight healthy donors were analyzed. Compared with non-primed neutrophils, the level of mPR3 expression was significantly higher on neutrophils primed with HMGB1 at concentration of 10 ng/ml (154.45 ± 60.55 vs. 274.71 ± 158.93, *P* = 0.023) (Figure 3A). Similarly, compared with non-primed neutrophils, the concentration of MPO was significantly higher in the supernatant of neutrophils primed with HMGB1 at concentration of 10 ng/ml (605.95 ± 183.86 ng/ml vs. 1,576.05 ± 878.55 ng/ml, *P* <0.001) (Figure 3C).

To exclude the influence of potentially contaminating platelet, we used three kinds of tubes containing different anticoagulant, that is, sodium citrate, EDTA and heparin, respectively, to collect blood and repeat our experiments. We found that the platelet contamination rates in neutrophils isolated from blood in EDTA tube and heparin tube were below 2%, which is generally considered to be acceptable [29]. Furthermore, we stimulated neutrophils isolated from each anticoagulant tube with HMGB1 at 10 ng/ml, and found no significant difference on mPR3 expression on these neutrophils (see Additional file 1 and Figure S1 in Additional file 2).

### ANCA induced HMGB1-primed neutrophils to respiratory burst and degranulation

**Figure 4:**
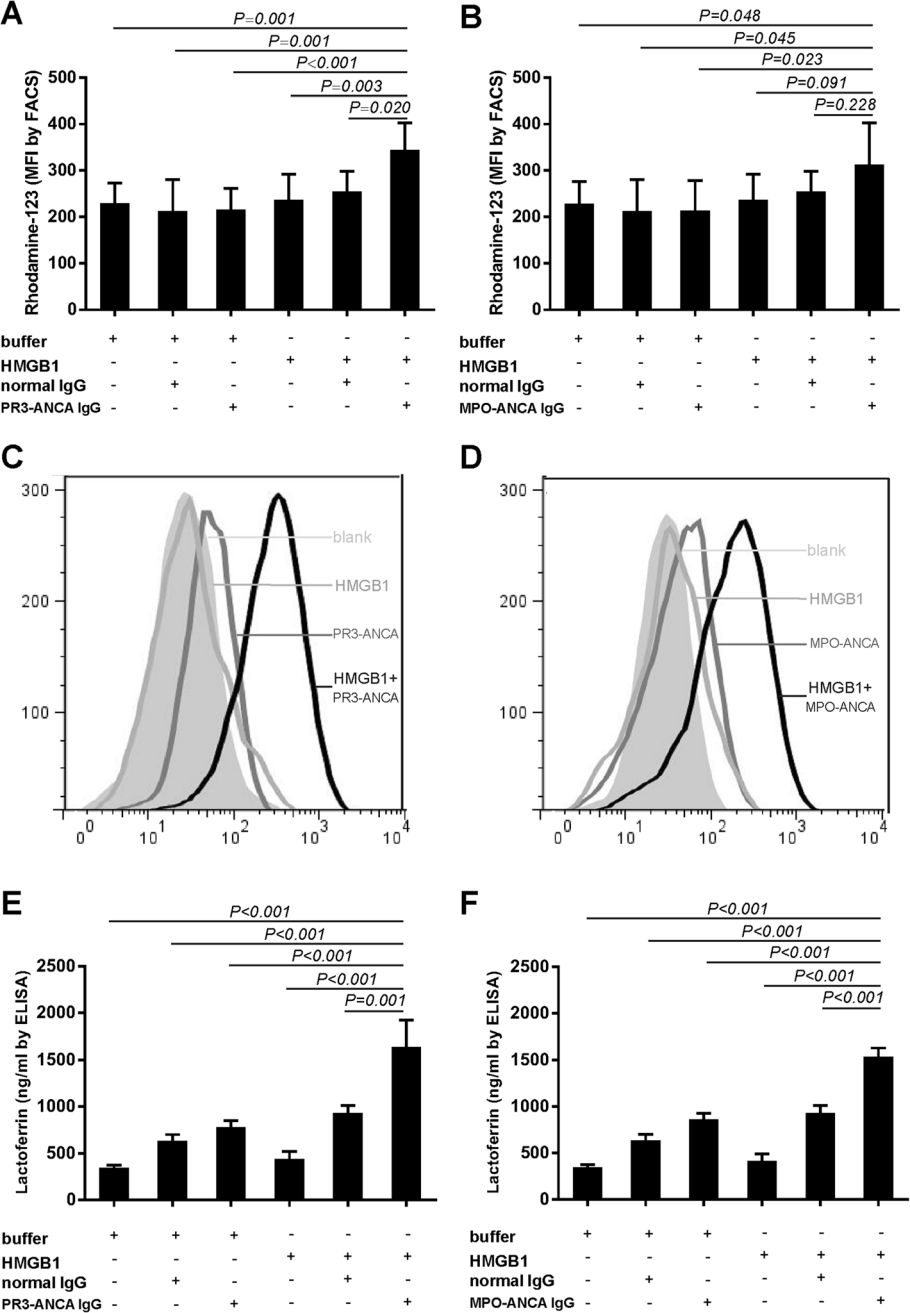
**Neutrophil respiratory burst and granulation induced by patient-derived PR3-ANCA-positive IgG or MPO-ANCA-positive IgG in HMGB1-primed neutrophils.** Neutrophil respiratory burst induced by patient-derived PR3-ANCA-positive IgG **(A)** or MPO-ANCA-positive IgG **(B)** was measured by conversion of dihydrorhodamine to rhodamine-123 in HMGB1-primed cells. **(C and** **D)** were representative histograms showing that PR3-ANCA-positive IgG and MPO-ANCA-positive IgG induced respiratory burst in HMGB1-primed neutrophils. Neutrophil granulation induced by patient-derived PR3-ANCA-positive IgG **(E)** or MPO-ANCA-positive IgG **(F)** was measured. Bars represent mean ± standard deviation (SD) of repeated measurements on neutrophils of 8 independent experiments and donors. All statistical differences between groups were shown in the Table S1 in Additional file 3. ANCA, antineutrophil cytoplasmic antibody; HMGB1, high mobility group box-1; IgG, immunoglobulin G; MPO, myeloperoxidase; PR3, proteinase 3.

We studied whether HMGB1 primed neutrophils for ANCA-induced respiratory burst. ANCA-IgG were prepared from two patients with active PR3-ANCA-positive vasculitis, two patients with active MPO-ANCA-positive vasculitis and two healthy volunteers, respectively. Neutrophils of the above-mentioned eight healthy donors were analyzed. Compared with non-primed neutrophils, the MFI value increased significantly in HMGB1-primed neutrophils activated with PR3-ANCA-positive IgG or MPO-ANCA-positive IgG (229.39 ± 43.56 vs. 343.92 ± 59.35, *P* = 0.001; 227.55 ± 48.44 vs. 312.89 ± 90.15, *P* = 0.048, respectively) (Figure 4A and B). No obvious respiratory burst activity was observed with HMGB1, ANCA-IgG alone, normal IgG alone or HMGB1 plus normal IgG, either.

Degranulation was determined by measuring the lactoferrin concentration in the supernatant of neutrophils stimulated by HMGB1 plus ANCA-positive IgGs. Compared with unstimulated neutrophils, the lactoferrin concentration increased significantly in the supernatant of HMGB1-primed neutrophils further induced by PR3-ANCA-positive IgG (345.77 ± 68.89 ng/ml vs. 1,639.88 ± 703.15 ng/ml, *P* <0.001) or MPO-ANCA- positive IgG (345.77 ± 68.89 ng/ml vs. 1,535.70 ± 230.59 ng/ml, *P* <0.001), respectively. Levels of degranulation were also increased in neutrophils incubated with ANCA-IgG alone, normal IgG alone or HMGB1 plus normal IgG to some degree. However, compared with the above neutrophils, the lactoferrin concentration still increased significantly in the supernatant of HMGB1-primed neutrophils further induced by PR3-ANCA-positive IgG or MPO-ANCA-positive IgG (*P* <0.001). There was no difference between neutrophils incubated with normal IgG alone and HMGB1 plus normal IgG (Figure 4E and F).

### ANCA antigens translocation, ANCA-induced respiratory burst and degranulation in HMGB1-primed neutrophils were TLR4- and RAGE-dependent

Result of a cytotoxicity assay showed that neutrophils first incubated with anti-TLR2 at 5 μg/ml, anti-TLR4 at 5 μg/ml, RAGE-Fc at 5 μM, MyD88 inhibitor at 20 μM or NF-κB inhibitor at 20 μM for 1 h on ice had same viability as those with buffer control for 1 h on ice (Figure 2A).

**Figure 5:**
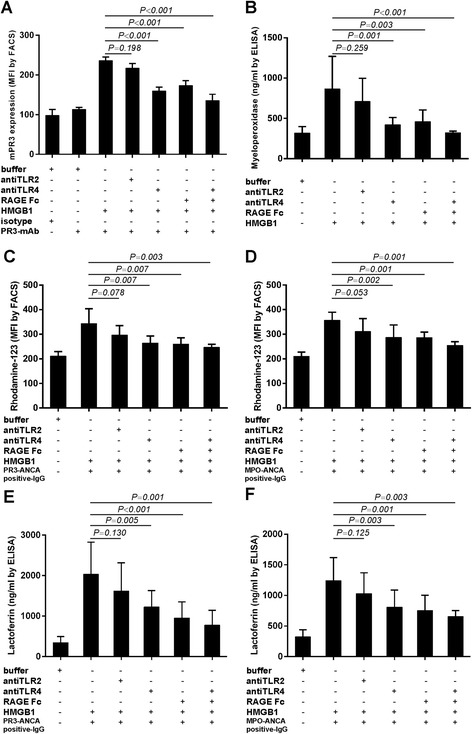
**Blockage of TLR4 and RAGE rather than TLR2 decreased ANCA antigens translocation, ANCA-induced respiratory burst and degranulation in HMGB1-primed neutrophils.** Blockage of TLR4 and RAGE rather than TLR2 decreased expression of mPR3 on HMGB1-primed neutrophils **(A)** and concentration of MPO in the neutrophils culture supernatant **(B)**. Blockage of TLR4 and RAGE rather than TLR2 decreased neutrophil respiratory burst induced by patient-derived PR3-ANCA-positive IgG **(C)** or MPO-ANCA-positive IgG **(D)** in HMGB1-primed cells. Blockage of TLR4 and RAGE rather than TLR2 decreased neutrophil degranulation induced by patient-derived PR3-ANCA-positive IgG **(E)** or MPO-ANCA-positive IgG **(F)** in HMGB1-primed cells. Bars represent mean ± standard deviation (SD) of repeated measurements on neutrophils of 8 independent experiments and donors. All statistical differences between groups were shown in the Table S2 in Additional file 3. ANCA, antineutrophil cytoplasmic antibody; HMGB1, high mobility group box-1; IgG, immunoglobulin G; MPO, myeloperoxidase; PR3, proteinase 3; RAGE, receptor for advanced glycation end products; TLR, Toll-like receptor.

Using flow cytometry, we showed that parallel experiments blocking TLR4 and RAGE resulted in a significant decrease in HMGB1-induced translocation of mPR3. mPR3 expression decreased from 236.15 ± 24.79 in HMGB1-primed neutrophils to 159.54 ± 22.96 by pre-incubating with anti-TLR4 antibody (*P* <0.001) or 173.28 ± 29.78 by pre-incubating with RAGE antagonist (*P* <0.001), while no significant decrease in neutrophils pre-incubating with anti-TLR2 antibody (236.15 ± 24.79 vs. 217.25 ± 26.53, *P* = 0.198) (Figure 5A). MPO concentration decreased from 864.65 ± 405.22 ng/ml in the supernatant of HMGB1-primed neutrophils to 421.02 ± 89.64 ng/ml by pre-incubating with anti-TLR4 antibody (*P* = 0.001) or 457.73 ± 146.74 ng/ml by pre-incubating with RAGE antagonist (*P* = 0.003, respectively), while no significant decrease was found in neutrophils pre-incubating with anti-TLR2 antibody (864.65 ± 405.22 ng/ml vs. 709.77 ± 287.92 ng/ml, *P* = 0.259) (Figure 5B).

Blocking TLR4 and RAGE rather than TLR2 also decreased oxygen radical production and lactoferrin release in HMGB1-primed neutrophils induced by ANCA-positive IgG from AAV patients. In HMGB1-primed neutrophils, subsequently activating with PR3-ANCA-positive IgG, the MFI value was 356.23 ± 33.21, which decreased to 286.48 ± 51.43 upon pre-incubating with anti-TLR4 antibody (*P* = 0.007), or 285.92 ± 22.80 by pre-incubating with RAGE antagonist (*P* = 0.007), while no significant decrease in neutrophils pre-incubating with anti-TLR2 antibody (356.23 ± 33.21 vs. 310.53 ± 52.80, *P* = 0.078) (Figure 5C). For MPO-ANCA-positive IgG, the MFI value was 343.08 ± 61.08, which decreased to 259.91 ± 25.66 by pre-incubating with anti-TLR4 antibody (*P* = 0.002) or 246.80 ± 12.30 by pre-incubating with RAGE antagonist (*P* = 0.001, respectively), while no significant decrease in neutrophils pre-incubating with anti-TLR2 antibody (343.08 ± 61.08 vs. 296.58 ± 38.42, *P* = 0.053) (Figure 5D). The lactoferrin concentration in the supernatant of HMGB1-primed neutrophils induced by PR3-ANCA-positive IgG decreased from 2,033.75 ± 791.81 ng/ml to 1,227.35 ± 400.57 ng/ml by pre-incubating with anti-TLR4 antibody (*P* = 0.005), or to 956.94 ± 394.23 by pre-incubating with RAGE antagonist (*P* <0.001), while no significant decrease in neutrophils pre-incubating with anti-TLR2 antibody (2,033.75 ± 791.81 ng/ml vs. 1,620.53 ± 698.98, *P* = 0.130) (Figure 5E). For MPO-ANCA-positive IgG, the lactoferrin concentration in the supernatant decreased from 1,242.20 ± 377.44 ng/ml in the supernatant of HMGB1-primed neutrophils induced by MPO-ANCA-positive IgG to 810.69 ± 278.61 ng/ml by pre-incubating with anti-TLR4 antibody (*P* = 0.003), or 754.56 ± 249.61 ng/ml by pre-incubating with RAGE antagonist (*P* = 0.001), while no significant decrease in neutrophils pre-incubating with anti-TLR2 antibody (1,242.20 ± 377.44 ng/ml vs. 1030.63 ± 339.24 ng/ml, *P* = 0.125) (Figure 5F).

Combined blockade of TLR4 and RAGE further decreased ANCA antigens translocation, ANCA-induced respiratory burst and degranulation in HMGB1-primed neutrophils to a certain degree. However, the effects were not completely overlaid (Figure 5).

To further confirm the receptors through which HMGB1 exerts its effects, we used TLR2−/− and TLR4−/− mice [30] and a neutrophil-like HL-60 cell line transfected by TLR2, TLR4 or RAGE siRNA to interfere the expression of corresponding receptors at genetic level to validate our findings. The results were in line with our data of human neutrophils with inhibitors and blocking antibodies to block the activity of corresponding receptors. For detailed information, see Additional file 4, Figure S2 in Additional file 5, Figure S3 in Additional file 6 and Figure S4 in Additional file 7.

### Enhanced activation of MyD88 and NF-κB contributed to the priming effect of HMGB1 on neutrophils

Since ANCA antigens translocation, ANCA-induced respiratory burst and degranulation in HMGB1-primed neutrophils were TLR4- and RAGE-dependent, we next investigated whether HMGB1-primed neutrophils were dependent on activation of MyD88/NF-κB pathways. We analyzed the effect of HMGB1 priming as well as the effect of the subsequent ANCA-positive IgG stimulation.

**Figure 6:**
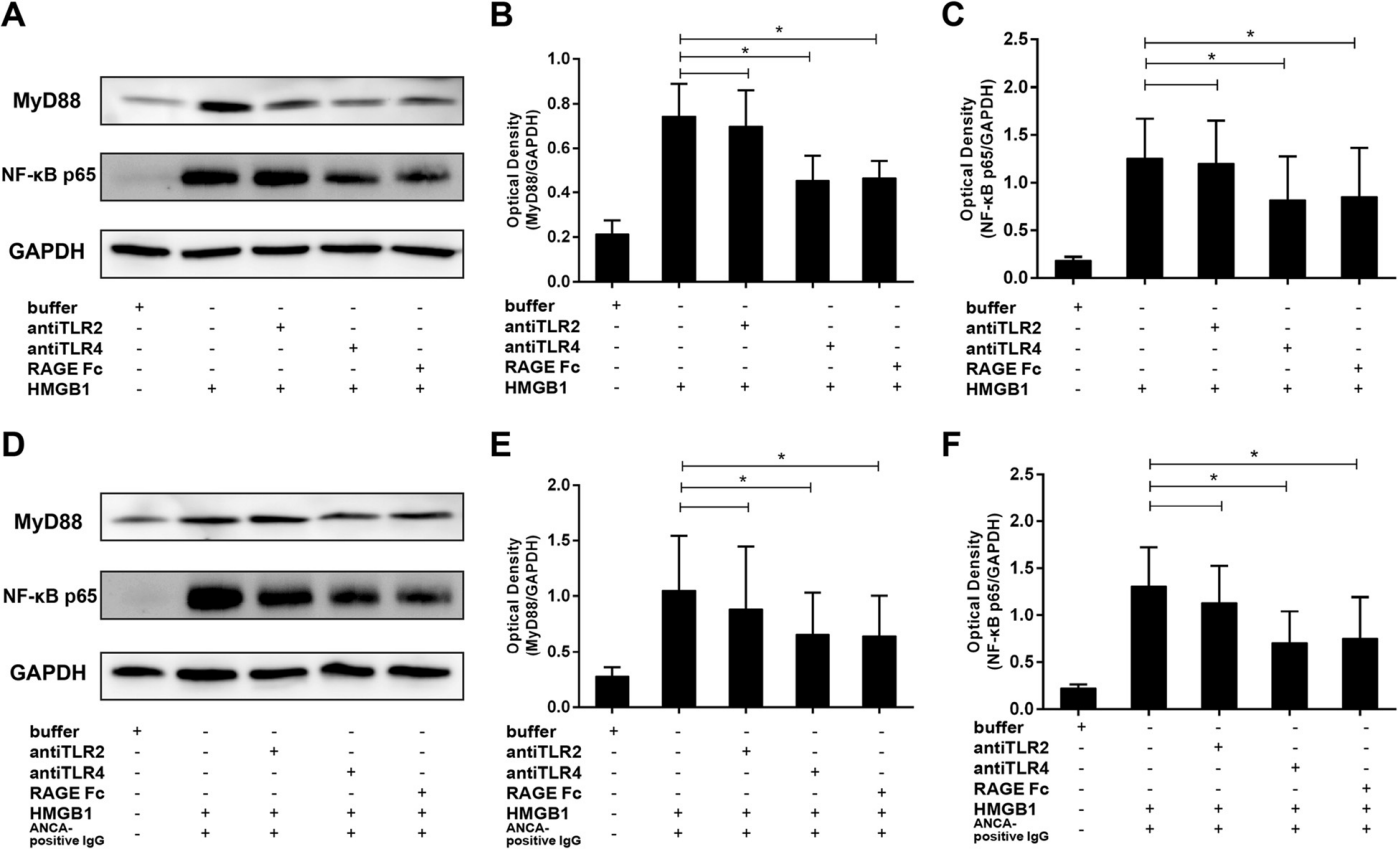
**Western blot analysis for MyD88 and phospho-NF-κ** **B.** Neutrophils were stimulated with HMGB1 10 ng/ml or buffer for 30 min, certain groups followed by stimulation with ANCA-positive IgG. Samples were harvested and MyD88 and phospho-NF-κB were determined by immunoblotting. A representative example is shown. GAPDH is shown as loading control **(A and** **D)**. The corresponding densitometric analysis was shown in **B**, **C**, **E** and **F** (n = 3). ^*^ *P* <0.05. Bars represent mean ± standard deviation (SD) of repeated measurements of 3 independent experiments. ANCA, antineutrophil cytoplasmic antibody; HMGB1, high mobility group box-1; IgG, immunoglobulin G; MyD88, myeloid differentiation factor 88; NF-κB, nuclear factor kappa B.

Western blot analysis was performed to study the expression of MyD88 and phosphorylated NF-κB p65. Figure 6 showed a representative Western blot analysis of MyD88 and phosphorylated NF-κB p65 and the corresponding Western blot analysis. We observed significantly increased MyD88 and phosphorylated NF-κB p65 in neutrophils incubated with HMGB1 or HMGB1 plus ANCA-positive IgG, which then decreased in certain groups by pre-incubating with anti-TLR4 antibody or RAGE antagonist. However, there are no significant change of the expression of MyD88 and phosphorylated NF-κB p65 in the group pre-incubated by anti-TLR2 antibody.

**Figure 7:**
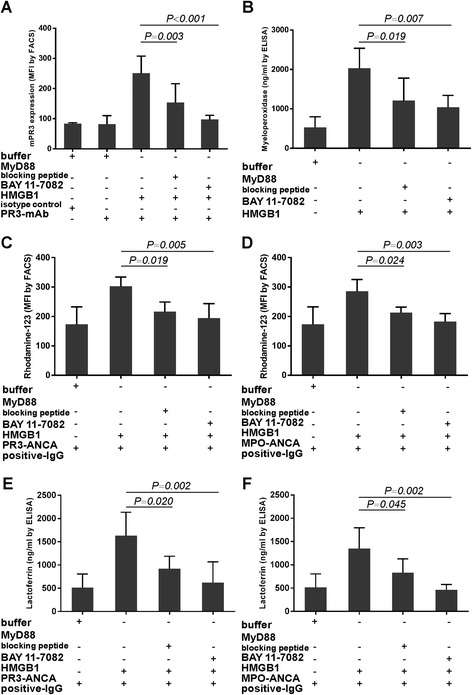
**MyD88 and NF-κ** **B inhibitors decreased ANCA antigens translocation, ANCA-induced respiratory burst and degranulation in HMGB1-primed neutrophils.** Inhibition of MyD88 and NF-κB decreased expression of mPR3 on HMGB1-primed neutrophils **(A)** and concentration of MPO in the neutrophils culture supernatant **(B)**. Inhibition of MyD88 and NF-κB decreased neutrophil respiratory burst induced by patient-derived PR3-ANCA-positive IgG **(C)** or MPO-ANCA-positive IgG **(D)** in HMGB1-primed cells. Inhibition of MyD88 and NF-κB decreased neutrophil degranulation induced by patient-derived PR3-ANCA-positive IgG **(E)** or MPO-ANCA-positive IgG **(F)** in HMGB1-primed cells. Bars represent mean ± standard deviation (SD) of repeated measurements on neutrophils of five independent experiments and donors. All statistical differences between groups were shown in the Table S3 in Additional file 3.

Compared with the neutrophils merely primed by HMGB1 and subsequently stimulated by ANCA-positive IgG, pre-incubation of neutrophils with inhibitors of MyD88 and NF-κB decreased ANCA antigens translocation, ANCA-induced respiratory burst and degranulation in HMGB1-primed neutrophils. mPR3 expression decreased by pre-incubating with inhibitors of MyD88 and NF-κB (251.25 ± 56.38 vs. 153.75 ± 62.61, *P* = 0.003; 251.25 ± 56.38 vs. 97.38 ± 13.68, *P* <0.001, respectively) (Figure 7A). Similarly, MPO concentration in the supernatant of neutrophils decreased by pre-incubating with inhibitors of MyD88 and NF-κB (2,032.47 ± 503.41 ng/ml vs. 1,207.38 ± 572.31 ng/ml, *P* = 0.019; 2,032.47 ± 503.41 ng/ml vs. 1,035.93 ± 304.65 ng/ml, *P* <0.001, respectively) (Figure 7B).

Blocking MyD88 or NF-κB also decreased oxygen radical production in HMGB1-primed neutrophils induced by ANCA-positive IgG from AAV patients (for PR3-ANCA-positive IgG, 302.40 ± 31.72 vs. 216.54 ± 32.63, *P* = 0.019; 302.40 ± 31.72 vs. 194.33 ± 49.07, *P* = 0.005, respectively; for MPO-ANCA-positive IgG, 285.24 ± 40.39 vs. 212.88 ± 18.61, *P* = 0.024; 285.24 ± 40.39 vs. 182.33 ± 27.46, *P* = 0.003, respectively) (Figure 7C and D).

Blocking MyD88 or NF-κB had the similar effect on degranulation in HMGB1-primed neutrophils induced by ANCA-positive IgG. The lactoferrin concentration in the supernatant decreased significantly (for PR3-ANCA-positive IgG, 1,633.21 ± 504.05 ng/ml vs. 920.55 ± 268.33 ng/ml, *P* = 0.02; 1,633.21 ± 504.05 ng/ml vs. 624.13 ± 444.27 ng/ml, *P* = 0.002, respectively; for MPO-ANCA-positive IgG, 1,350.15 ± 447.05 ng/ml vs. 832.80 ± 295.00 ng/ml, *P* = 0.045; 1,350.15 ± 447.05 ng/ml vs. 460.97 ± 119.93 ng/ml, *P* = 0.002, respectively) (Figure 7E and F). These data suggested important roles for the TLR4/MyD88/NF-κB pathway and RAGE/MyD88/NF-κB pathway in HMGB1-mediated priming of neutrophils.

## Discussion

As an essential DAMP molecule, HMGB1 is released by stimulated neutrophils, macrophages and monocytes, and then regulates cytokine expression and promotes inflammatory cell recruitment [31,32]. Furthermore, elevated levels of HMGB1 have been implicated in the pathogenesis of a broad range of acute and chronic inflammatory conditions in sepsis, cancer, systemic lupus erythematosus and rheumatoid arthritis [33-37]. Recently, we observed circulating HMGB1 levels are associated with the disease activity of AAV [14], which was in line with and further extended the earlier related findings [38-40], although it remains controversial in studies by de Souza *et al*. [41,42]. Whether HMGB1 participated in the pathogenesis of AAV, and then the underlying mechanism was not fully clear yet.

ANCA-induced neutrophils respiratory burst and degranulation was crucial in the development of AAV, with assistance from a variety of proinflammatory factors by priming the neutrophils to express more ANCA target antigens [2-4]. Taking the multiple effects of HMGB1 on neutrophils into consideration, we carried out the investigation to figure out the role of HMGB1 in ANCA-induced neutrophil activation. The results showed that HMGB1 is able to prime neutrophils by increasing ANCA antigens translocation, and the primed neutrophils could be further activated by ANCA-positive IgG from AAV patients, resulting in the respiratory burst and degranulation.

According to our previous studies [21], the increase in membrane-bound MPO expression were much lower than that in mPR3 expression after incubation with priming factors, which was in line with some other studies [6,23]. Therefore, in the current study, we measured the concentrations of secreted MPO in the supernatant of HMGB1-primed neutrophils using specific ELISA kit. However, it was inconsistent with Maugeri’s study [43], in which neutrophils express more membrane-bound MPO after stimulated by HMGB1. Such discrepancy might be attributed to the difference of incubation time. In Maugeri’s study, neutrophils were incubated with HMGB1 for 5 minutes, which was much shorter than that in our current study (30 minutes). As reported by Witko-Sarsat *et al.* [22], MPO was mainly released into the extracellular medium and PR3 was released in minute amounts into the extracellular medium. It was possible that the membrane-bound MPO had been released into the extracellular medium when we measured the neutrophils. It is also notable that, compared with neutrophils incubated with HMGB1 alone, the MPO concentration increased significantly in the supernatant of HMGB1-primed neutrophils further activated by ANCA-positive IgG (see Figure S5 in Additional file 8). It indicated the above process of MPO releasing from neutrophils incubated with HMGB1 alone is a priming process, rather than an actual complete degranulation with granule depletion.

HMGB1 exerts its proinflammatory effects through various receptors, among which, RAGE, TLR2 and TLR4 on the neutrophils surface may be the representative ones [11-13]. TLR4 transmits signals *via* MyD88-dependent and MyD88-independent pathways, and the former signaling pathway, which might finally activate NF-κB, was shown to be responsible for proinflammatory cytokine expression [27,44,45]. RAGE engagement by multiple ligands activates various signaling pathways, that is, through the adaptors Dia-1, TIR domain-containing adaptor protein (TIRAP)-MyD88 and/or as yet unidentified adaptors, which in turn activate signaling molecules impinging on transcription factors, such as NF-κB. Besides, activation of RAGE induces generation of oxygen radicals by a yet unknown mechanism [28,46]. In the current study, ANCA antigens translocation, ANCA-induced respiratory burst and degranulation in HMGB1-primed neutrophils were found to be TLR4- and RAGE-dependent. Moreover, the signaling pathways of both TLR4 and RAGE might be MyD88-dependent and finally activate NF-κB, causing its phosphorylation. To some extent, the inhibitory effect by blocking receptors and signaling molecules on ANCA-induced activation of HMGB1-primed neutrophils might be through inhibition of ANCA target antigens translocation. We also found combined blockade of TLR4 and RAGE could further decrease the effects of HMGB1 on neutrophils. However, these effects were not completely overlaid. We speculated that there are some crosstalks between these two receptors and they may share partial pathways involved in this process. What is more, the effects of HMGB1 exerts on neutrophils may be dose-dependent, mainly through the TLR4- and RAGE-MyD88/NF-κB pathway. For example, a lower concentration of HMGB1 at 5 ng/ml, which was comparable to the circulating HMGB1 level in AAV patients at remission stage [14], still depended on the same pathways to exert the effects on neutrophils (see Figure S6 in Additional file 9).

## Conclusions

Our data demonstrated that HMGB1 is able to prime neutrophils by increasing ANCA antigens translocation, and the primed neutrophils could be further induced by ANCA, resulting in the respiratory burst and degranulation. The process is TLR4- and RAGE-dependent through the MyD88/NF-κB pathway. It suggested that blockade of HMGB1 might limit inflammatory damage caused by ANCA-activated neutrophils, and figuring out the signaling pathways in the process might provide potential clues for intervention strategies.

## Additional files

Additional file 1:
**To evaluate the influence of contaminating platelet in neutrophils isolated from different anticoagulant tubes.**
Additional file 2: Figure S1. The platelet contamination rates and membrane-bound PR3 expression on these neutrophils after priming in neutrophils isolated from blood in sodium citrate tube, EDTA tube and heparin tube. The platelet contamination rates in neutrophils isolated from blood in sodium citrate tube **(A)**, EDTA tube **(B)** and heparin tube **(C)**. The membrane-bound PR3 expression on these neutrophils after priming in neutrophils isolated from blood in sodium citrate tube **(D)**, EDTA tube **(E)** and heparin tube **(F)**. Additional file 3: Table S1. Statistical differences between groups in Figure 4. **Table S2.** Statistical differences between groups in Figure 5. **Table S3.** Statistical differences between groups in Figure 7. Additional file 4:
**To confirm the receptors of HMGB1 by gene knockout animals and a neutrophil-like HL-60 cell line transfected with small interfering RNAs.**
Additional file 5: Figure S2. Release of MPO by HMGB1-primed murine neutrophils from TLR2−/− and TLR4−/− mice. HMGB1 increased concentration of MPO in the culture supernatant of neutrophils from TLR2 −/− mice as wild-type mice **(A)**. HMGB1 could not increase concentration of MPO in the culture supernatant of neutrophils from TLR4 −/− mice as wild-type mice **(B)**. Bars represent mean ± SD of repeated measurements on neutrophils of five independent experiments and mice. Additional file 6: Figure S3. Anti-MPO IgGs-induced respiratory burst in HMGB1-primed murine neutrophils from TLR2−/− and TLR4−/− mice. Murine neutrophil respiratory burst induced by anti-MPO IgGs was measured by conversion of dihydrorhodamine to Rho-123 in HMGB1-primed cells **(C, F)**. **A**-**B** and **D**-**E** were representative flow cytometry results. The percentage of Ly6G + Rho + neutrophils were regarded as level of respiratory burst. Bars represent mean ± SD of repeated measurements on neutrophils of three independent experiments and mice. Additional file 7: Figure S4. Expression of membrane-bound PR3 on HMGB1-primed neutrophils-like HL-60 cells and anti-MPO IgGs-induced respiratory burst in HMGB1-primed neutrophils-like HL-60 cells transfected with TLR2, TLR4 or RAGE siRNA. HMGB1 increased expression of membrane-bound PR3 on neutrophil-like HL-60 cells transfected with TLR2, TLR4 or RAGE siRNA **(A)**. Anti-MPO IgGs-induced respiratory burst in HMGB1-primed neutrophil-like HL-60 cells transfected with TLR2, TLR4 or RAGE siRNA **(B)**. Bars represent mean ± SD of repeated measurements on neutrophils of three independent experiments. Additional file 8: Figure S5. The MPO concentration in the supernatant of neutrophils under various stimulation. The MPO concentration increased significantly in the supernatant of HMGB1-primed neutrophils further activated by ANCA-positive IgG. Additional file 9: Figure S6. Expression of membrane-bound PR3 and anti-MPO IgGs-induced respiratory burst on neutrophils primed by a lower concentration of HMGB1 at 5 ng/ml with/without blockage of TLR2, TLR4, RAGE, MyD88 or NF-κB. The lower concentration of HMGB1 at 5 ng/ml still depended on the same pathways to exert the effects on neutrophils.

## Abbreviations

AAV: ANCA-associated vasculitis
ANCA: antineutrophil cytoplasmic antibody
BSA: bovine serum albumin
DAMP: damage-associated molecular pattern
EGPA: eosinophilic granulomatosis with polyangiits
ELISA: enzyme-linked immunosorbent assay
GPA: granulomatosis with polyangiitis
HBSS: Hank’s balanced salt solution
HMGB1: high mobility group box-1
IgG: immunoglobulin G
IRAKs: interleukin-1 receptor-associated kinases
LDH: lactate dehydrogenase
MAPKK: mitogen-activated protein kinase-kinase
MFI: mean fluorescence intensity
MPA: microscopic polyangiitis
MPO: myeloperoxidase
MyD88: myeloid differentiation factor 88
NF-κB: nuclear factor kappa B
PE: phycoerythrin
PR3: proteinase 3
RAGE: receptor for advanced glycation end products
TBST: Tris-buffered saline with Tween 20
TIRAP: TIR domain-containing adaptor protein
TLR: Toll-like receptor
TNF-α: tumor necrosis factor alpha
TRAF6/IKK: TNF receptor-associated factor 6/IκB kinase
