# Supplementary material for: High mobility group box 1 contributes to anti-neutrophil cytoplasmic antibody-induced neutrophils activation through receptor for advanced glycation end products (RAGE) and Toll-like receptor 4
Source: Arthritis Res Ther. 2015 Mar 18;17(1):64. doi: 10.1186/s13075-015-0587-4 (PMC4382936; doi:10.1186/s13075-015-0587-4)
Supplement: Additional file 1: — To evaluate the influence of contaminating platelet in neutrophils isolated from different anticoagulant tubes. [file 13075_2015_587_MOESM1_ESM.doc]

**Additional file 1**

**To evaluate the influence of contaminating platelet in neutrophils isolated from different anticoagulant tubes**

**Materials and Methods**

**Reagents**

Recombinant human HMGB1 proteins were purchased from R&D Systems (C23-C45 disulfide C106 thiol form) (Abingdon, UK). FITC-conjugated mouse anti-human PR3 was purchased from Abcam (Cambridge, UK), with addition of irrelevant IgG control antibody. APC-conjugated mouse anti-human CD41 was purchased from BioLegend (San Diego, USA), with addition of irrelevant IgG control antibody.

**Neutrophil isolation**

Fresh blood of healthy donors was collected by three kinds of tubes containing different anticoagulant, i.e., sodium citrate, EDTA and heparin, respectively. The later procedure was as described in the manuscript.

**Measurement of** **platelet contamination rate in neutrophils isolated from different anticoagulant tubes**

Purified neutrophils were incubated with isotope control or APC-conjugated CD41, which is a platelet marker [1, 2], for 30min on ice. Flow cytometry was used to evaluate platelet contamination rate in these neutrophils. Platelets were gated in sideward scatter (SSC)/FL-4 mode (representing the level of APC-CD41).

**Measurement of membrane expression of membrane-bound PR3 on neutrophils isolated from different anticoagulant tubes after priming**

Flow cytometry was used to evaluate membrane-bound PR3 expression on neutrophils isolated from blood collected by sodium citrate tube, EDTA tube or heparin tube. Cells were incubated with HMGB1 or buffer control for 30 min at 37°C. All further steps were performed on ice and washing steps were carried out using HBSS +/+ containing 1% bovine serum albumin (BSA). TruStain fcX (BioLegend, San Diego, USA) was used in all samples prior to the addition of antibodies to block nonspecific antibodies binding. Next, cells were stained with a saturating dose of FITC-PR3 or with an irrelevant IgG1 control antibody for 30 min. Fluorescence intensity of FITC was analyzed using flow cytometry assessment of ANCA-antigen expression. Samples were analyzed using Accuri C6. The level of membrane-bound PR3-expression was calculated as mean fluorescence intensity (MFI) values.

**Result**

As shown in Additional file 4: Figure S1A-C, the platelet contamination rates in neutrophils isolated from blood in EDTA tube and heparin tube were below 2%, which is generally considered to be acceptable . The platelet contamination rate in cells isolated from blood in sodium citrate tube was above 2%.

Neutrophils isolated from each anticoagulant tube were primed with HMGB1 at 10ng/ml, and there was no significant difference on membrane-bound PR3 expression on these neutrophils (Additional file 4: Figure S1D-F).

**References**

1. Alonzo MT, Lacuesta TL, Dimaano EM, Kurosu T, Suarez LA, Mapua CA, Akeda Y, Matias RR, Kuter DJ, Nagata S *et al*: **Platelet apoptosis and apoptotic platelet clearance by macrophages in secondary dengue virus infections**. *J Infect Dis* 2012;**205**:1321-1329.

2. Coppinger JA, Cagney G, Toomey S, Kislinger T, Belton O, McRedmond JP, Cahill DJ, Emili A, Fitzgerald DJ, Maguire PB: **Characterization of the proteins released from activated platelets leads to localization of novel platelet proteins in human atherosclerotic lesions**. *Blood* 2004;**103**:2096-2104.

3. Ritis K, Doumas M, Mastellos D, Micheli A, Giaglis S, Magotti P, Rafail S, Kartalis G, Sideras P, Lambris JD: **A novel C5a receptor-tissue factor cross-talk in neutrophils links innate immunity to coagulation pathways**. *J Immunol* 2006;**177**:4794-4802.

**Figure legends**

**Additional file 4: Figure S1. The platelet contamination rates and membrane-bound PR3 expression on these neutrophils after priming in neutrophils isolated from blood in sodium citrate tube, EDTA tube and heparin tube.**

The platelet contamination rates in neutrophils isolated from blood in sodium citrate tube (A), EDTA tube (B) and heparin tube (C).

The membrane-bound PR3 expression on these neutrophils after priming in neutrophils isolated from blood in sodium citrate tube (D), EDTA tube (E) and heparin tube (F).
