# Supplementary figures and images for: High mobility group box 1 contributes to anti-neutrophil cytoplasmic antibody-induced neutrophils activation through receptor for advanced glycation end products (RAGE) and Toll-like receptor 4
Source: Arthritis Res Ther. 2015 Mar 18;17(1):64. doi: 10.1186/s13075-015-0587-4 (PMC4382936; doi:10.1186/s13075-015-0587-4)

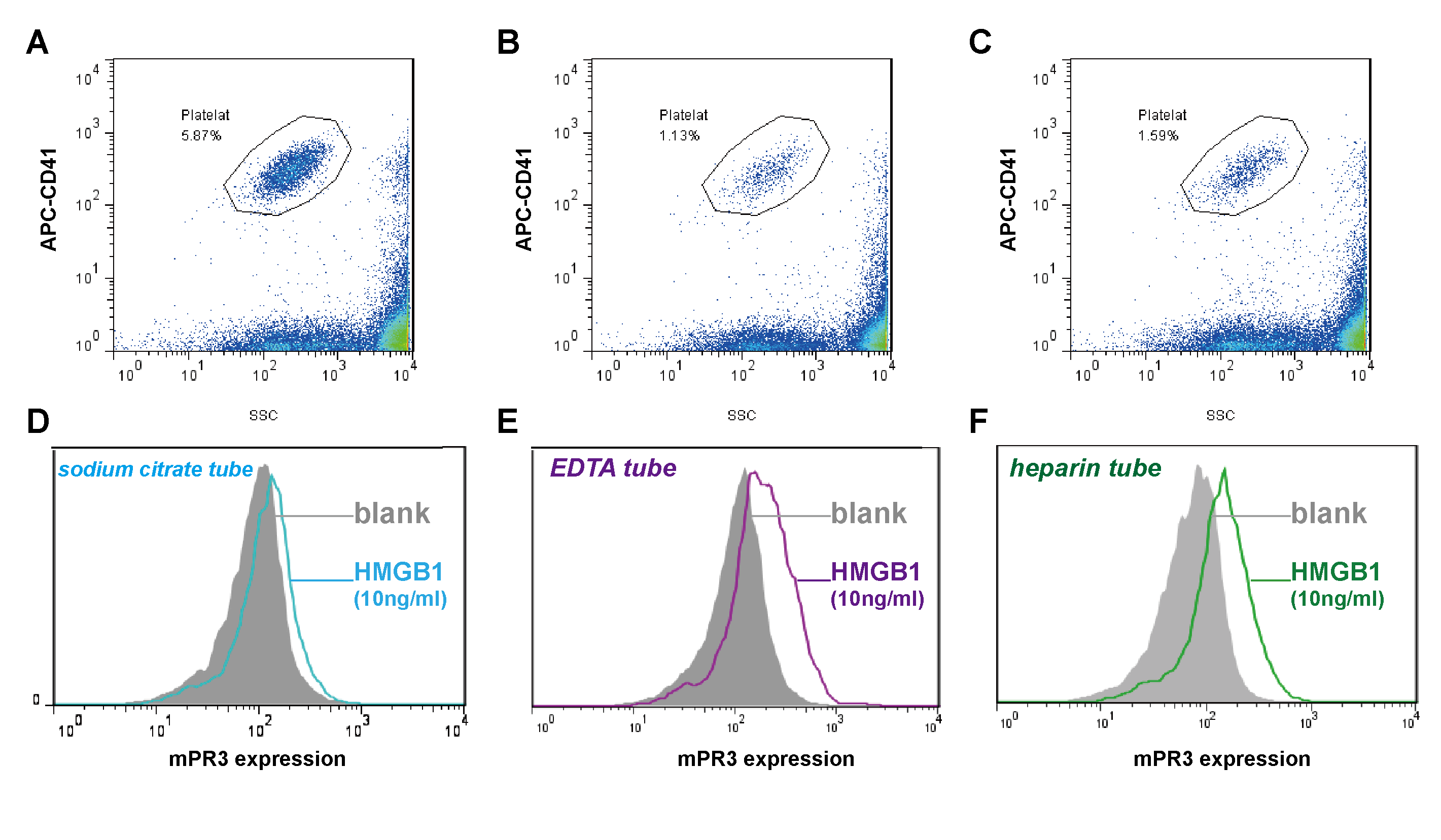

Supplement: Additional file 2: Figure S1. — The platelet contamination rates and membrane-bound PR3 expression on these neutrophils after priming in neutrophils isolated from blood in sodium citrate tube, EDTA tube and heparin tube. The platelet contamination rates in neutrophils isolated from blood in sodium citrate tube (A), EDTA tube (B) and heparin tube (C). The membrane-bound PR3 expression on these neutrophils after priming in neutrophils isolated from blood in sodium citrate tube (D), EDTA tube (E) and heparin tube (F). [file 13075_2015_587_MOESM2_ESM.tiff]

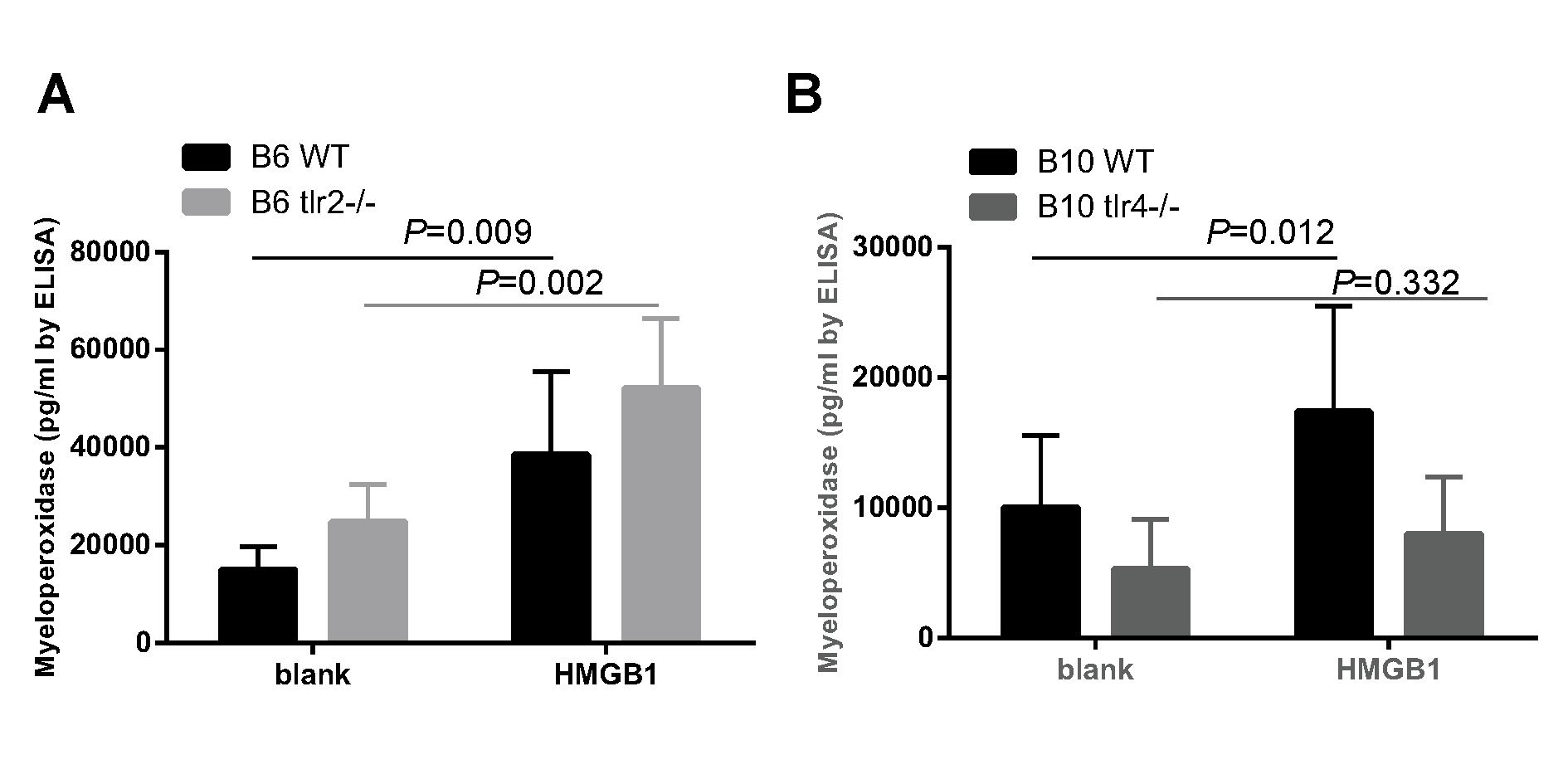

Supplement: Additional file 5: Figure S2. — Release of MPO by HMGB1-primed murine neutrophils from TLR2−/− and TLR4−/− mice. HMGB1 increased concentration of MPO in the culture supernatant of neutrophils from TLR2 −/− mice as wild-type mice (A). HMGB1 could not increase concentration of MPO in the culture supernatant of neutrophils from TLR4 −/− mice as wild-type mice (B). Bars represent mean ± SD of repeated measurements on neutrophils of five independent experiments and mice. [file 13075_2015_587_MOESM5_ESM.tiff]

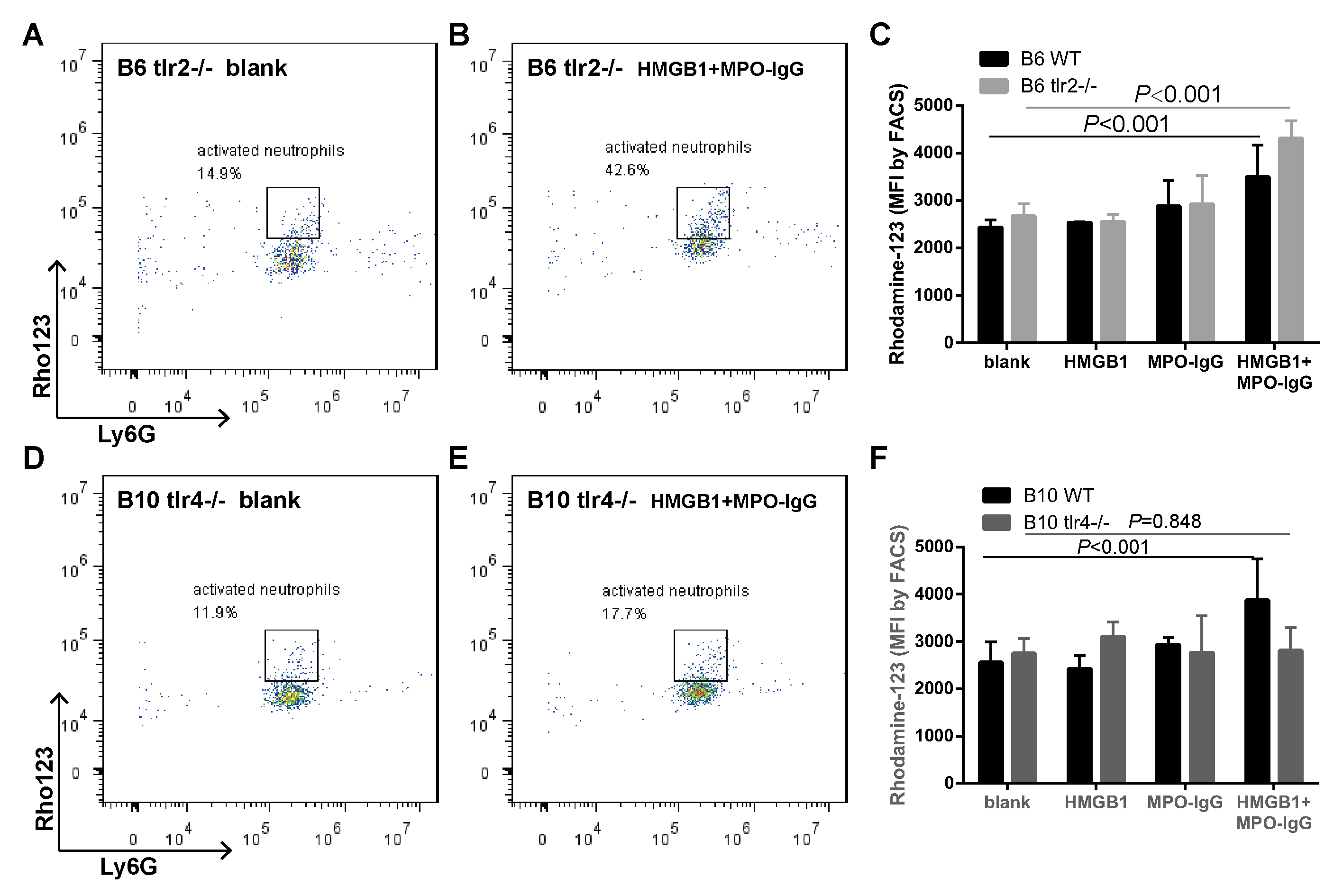

Supplement: Additional file 6: Figure S3. — Anti-MPO IgGs-induced respiratory burst in HMGB1-primed murine neutrophils from TLR2−/− and TLR4−/− mice. Murine neutrophil respiratory burst induced by anti-MPO IgGs was measured by conversion of dihydrorhodamine to Rho-123 in HMGB1-primed cells (C, F). A-B and D-E were representative flow cytometry results. The percentage of Ly6G + Rho + neutrophils were regarded as level of respiratory burst. Bars represent mean ± SD of repeated measurements on neutrophils of three independent experiments and mice. [file 13075_2015_587_MOESM6_ESM.tiff]

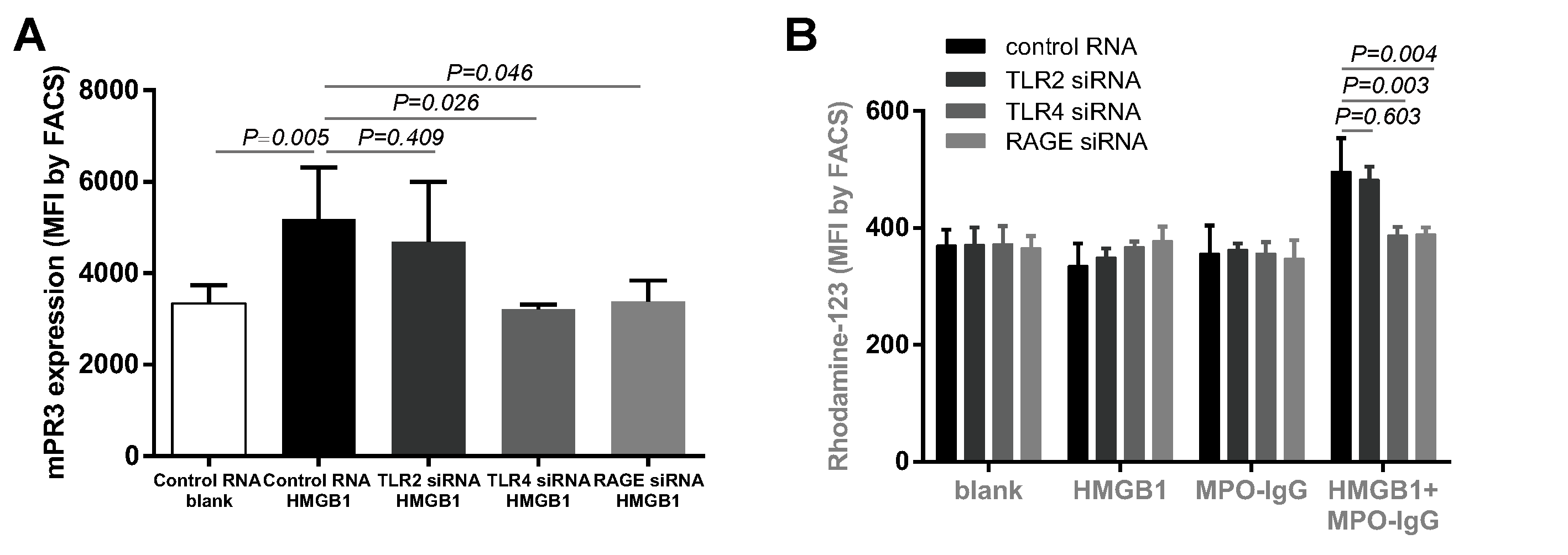

Supplement: Additional file 7: Figure S4. — Expression of membrane-bound PR3 on HMGB1-primed neutrophils-like HL-60 cells and anti-MPO IgGs-induced respiratory burst in HMGB1-primed neutrophils-like HL-60 cells transfected with TLR2, TLR4 or RAGE siRNA. HMGB1 increased expression of membrane-bound PR3 on neutrophil-like HL-60 cells transfected with TLR2, TLR4 or RAGE siRNA (A). Anti-MPO IgGs-induced respiratory burst in HMGB1-primed neutrophil-like HL-60 cells transfected with TLR2, TLR4 or RAGE siRNA (B). Bars represent mean ± SD of repeated measurements on neutrophils of three independent experiments. [file 13075_2015_587_MOESM7_ESM.tiff]

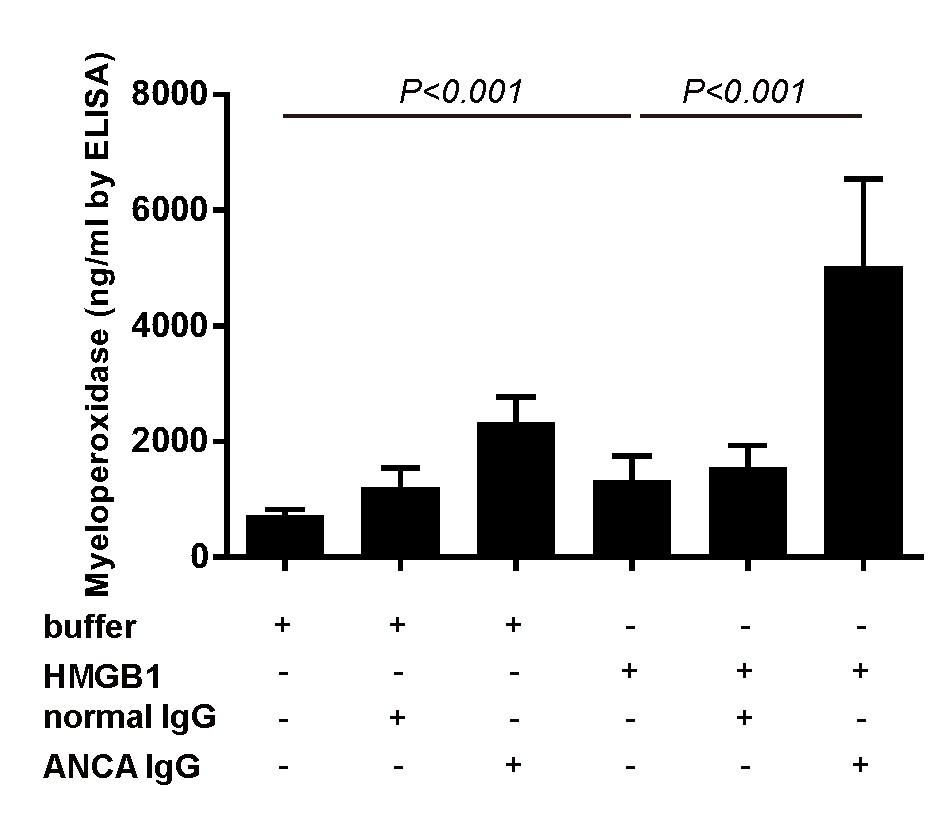

Supplement: Additional file 8: Figure S5. — The MPO concentration in the supernatant of neutrophils under various stimulation. The MPO concentration increased significantly in the supernatant of HMGB1-primed neutrophils further activated by ANCA-positive IgG. [file 13075_2015_587_MOESM8_ESM.tiff]

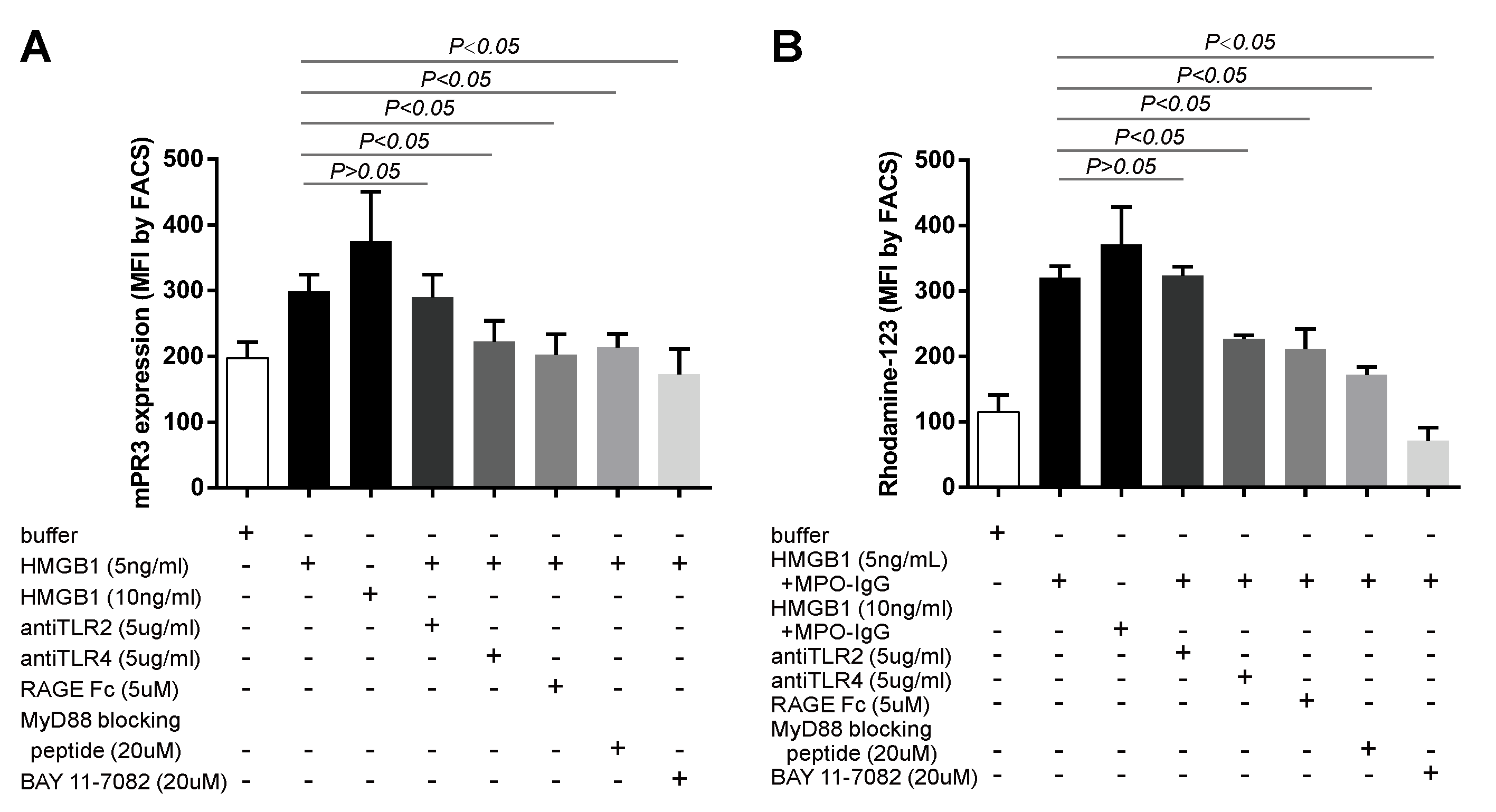

Supplement: Additional file 9: Figure S6. — Expression of membrane-bound PR3 and anti-MPO IgGs-induced respiratory burst on neutrophils primed by a lower concentration of HMGB1 at 5 ng/ml with/without blockage of TLR2, TLR4, RAGE, MyD88 or NF-κB. The lower concentration of HMGB1 at 5 ng/ml still depended on the same pathways to exert the effects on neutrophils. [file 13075_2015_587_MOESM9_ESM.tiff]
