# Supplementary material for: High mobility group box 1 contributes to anti-neutrophil cytoplasmic antibody-induced neutrophils activation through receptor for advanced glycation end products (RAGE) and Toll-like receptor 4
Source: Arthritis Res Ther. 2015 Mar 18;17(1):64. doi: 10.1186/s13075-015-0587-4 (PMC4382936; doi:10.1186/s13075-015-0587-4)
Supplement: Additional file 3: Table S1. — Statistical differences between groups in Figure 4. Table S2. Statistical differences between groups in Figure 5. Table S3. Statistical differences between groups in Figure 7. [file 13075_2015_587_MOESM3_ESM.doc]

**Additional file 3**

**Table S1. Statistical differences between groups in Figure 4**

A

|  | blank | normal IgG | PR3-ANCA IgG | HMGB1 | HMGB1+normal IgG | HMGB1+PR3-ANCA |
| --- | --- | --- | --- | --- | --- | --- |
| blank |  | .639 | .634 | .837 | .511 | .001 |
| normal IgG | .639 |  | .923 | .544 | .332 | .001 |
| PR3-ANCA IgG | .634 | .923 |  | .527 | .294 | .000 |
| HMGB1 | .837 | .544 | .527 |  | .662 | .003 |
| HMGB1+normal IgG | .511 | .332 | .294 | .662 |  | .020 |
| HMGB1+PR3-ANCA | .001 | .001 | .000 | .003 | .020 |  |

B

|  | blank | normal IgG | MPO-ANCA IgG | HMGB1 | HMGB1+normal IgG | HMGB1+MPO-ANCA | |
| --- | --- | --- | --- | --- | --- | --- | --- |
| blank |  | .751 | .742 | .845 | .592 | | .048 |
| normal IgG | .751 |  | .974 | .635 | .447 | | .045 |
| MPO-ANCA IgG | .742 | .974 |  | .614 | .415 | | .023 |
| HMGB1 | .845 | .635 | .614 |  | .732 | | .091 |
| HMGB1+normal IgG | .592 | .447 | .415 | .732 |  | | .228 |
| HMGB1+MPO-ANCA | .048 | .045 | .023 | .091 | .228 | |  |

E

|  | blank | normal IgG | PR3-ANCA IgG | HMGB1 | HMGB1+normal IgG | HMGB1+PR3-ANCA |
| --- | --- | --- | --- | --- | --- | --- |
| blank |  | .141 | .037 | .643 | .005 | .000 |
| normal IgG | .141 |  | .466 | .338 | .134 | .000 |
| PR3-ANCA IgG | .037 | .466 |  | .112 | .471 | .000 |
| HMGB1 | .643 | .338 | .112 |  | .021 | .000 |
| HMGB1+normal IgG | .005 | .134 | .471 | .021 |  | .001 |
| HMGB1+PR3-ANCA | .000 | .000 | .000 | .000 | .001 |  |

F

|  | blank | normal IgG | MPO-ANCA IgG | HMGB1 | HMGB1+normal IgG | HMGB1+MPO-ANCA |
| --- | --- | --- | --- | --- | --- | --- |
| blank |  | .007 | .000 | .566 | .000 | .000 |
| normal IgG | .007 |  | .070 | .081 | .006 | .000 |
| MPO-ANCA IgG | .000 | .070 |  | .004 | .589 | .000 |
| HMGB1 | .566 | .081 | .004 |  | .000 | .000 |
| HMGB1+normal IgG | .000 | .006 | .589 | .000 |  | .000 |
| HMGB1+MPO-ANCA | .000 | .000 | .000 | .000 | .000 |  |

**Table S2. Statistical differences between groups in Figure 5**

A

|  | isotype | blank | HMGB1 | antiTLR2+HMGB1 | antiTLR4+HMGB1 | RAGE Fc+HMGB1 | anti4+R+HMGB1 |
| --- | --- | --- | --- | --- | --- | --- | --- |
| isotype |  | .318 | .000 | .000 | .000 | .000 | .084 |
| blank | .318 |  | .000 | .000 | .002 | .000 | .272 |
| HMGB1 | .000 | .000 |  | .198 | .000 | .000 | .000 |
| antiTLR2+HMGB1 | .000 | .000 | .198 |  | .001 | .007 | .001 |
| antiTLR4+HMGB1 | .000 | .002 | .000 | .001 |  | .352 | .253 |
| RAGE Fc+HMGB1 | .000 | .000 | .000 | .007 | .352 |  | .076 |
| anti4+R+HMGB1 | .084 | .272 | .000 | .001 | .253 | .076 |  |

B

|  | blank | HMGB1 | antiTLR2+HMGB1 | antiTLR4+HMGB1 | RAGE Fc+HMGB1 | anti4+R+HMGB1 |
| --- | --- | --- | --- | --- | --- | --- |
| blank |  | .064 | .446 | .138 | .243 | .043 |
| HMGB1 | .064 |  | .259 | .001 | .003 | .000 |
| antiTLR2+HMGB1 | .446 | .259 |  | .024 | .052 | .007 |
| antiTLR4+HMGB1 | .138 | .001 | .024 |  | .741 | .416 |
| RAGE Fc+HMGB1 | .243 | .003 | .052 | .741 |  | .281 |
| anti4+R+HMGB1 | .043 | .000 | .007 | .416 | .281 |  |

C

|  | blank | HMGB1  +PR3-ANCA | antiTLR2+HMGB1+PR3-ANCA | antiTLR4+HMGB1+PR3-ANCA | RAGE Fc+HMGB1  +PR3-ANCA | | anti4+R+HMGB1+PR3-ANCA |
| --- | --- | --- | --- | --- | --- | --- | --- |
| blank |  | .000 | .001 | .004 | .004 | .162 | |
| HMGB1+PR3-ANCA | .000 |  | .078 | .007 | .007 | .003 | |
| antiTLR2+HMGB1+PR3-ANCA | .001 | .078 |  | .340 | .329 | .092 | |
| antiTLR4+HMGB1+PR3-ANCA | .004 | .007 | .340 |  | .981 | .308 | |
| RAGE Fc+HMGB1+PR3-ANCA | .004 | .007 | .329 | .981 |  | .316 | |
| anti4+R+HMGB1+PR3-ANCA | .162 | .003 | .092 | .308 | .316 |  | |

D

|  | blank | HMGB1  +MPO-ANCA | antiTLR2+HMGB1+MPO-ANCA | antiTLR4+HMGB1+MPO-ANCA | RAGE Fc+HMGB1  +MPO-ANCA | | anti4+R+HMGB1+MPO-ANCA |
| --- | --- | --- | --- | --- | --- | --- | --- |
| blank |  | .000 | .001 | .029 | .043 | .187 | |
| HMGB1+ANCA | .000 |  | .053 | .002 | .001 | .001 | |
| antiTLR2+HMGB1+MPO-ANCA | .001 | .053 |  | .168 | .121 | .071 | |
| antiTLR4+HMGB1+MPO-ANCA | .029 | .002 | .168 |  | .855 | .517 | |
| RAGE Fc+HMGB1+MPO-ANCA | .043 | .001 | .121 | .855 |  | .623 | |
| anti4+R+HMGB1+MPO-ANCA | .187 | .001 | .071 | .517 | .623 |  | |

E

|  | blank | HMGB1  +PR3-ANCA | antiTLR2+HMGB1+PR3-ANCA | antiTLR4+HMGB1+PR3-ANCA | RAGE Fc+HMGB1  +PR3-ANCA | | anti4+R+HMGB1+PR3-ANCA |
| --- | --- | --- | --- | --- | --- | --- | --- |
| blank |  | .000 | .000 | .001 | .019 | .212 | |
| HMGB1+PR3-ANCA | .000 |  | .130 | .005 | .000 | .001 | |
| antiTLR2+HMGB1+PR3-ANCA | .000 | .130 |  | .136 | .014 | .021 | |
| antiTLR4+HMGB1+PR3-ANCA | .001 | .005 | .136 |  | .301 | .208 | |
| RAGE Fc+HMGB1+PR3-ANCA | .019 | .000 | .014 | .301 |  | .615 | |
| anti4+R+HMGB1+PR3-ANCA | .212 | .001 | .021 | .208 | .615 |  | |

F

|  | blank | HMGB1  +MPO-ANCA | antiTLR2+HMGB1+MPO-ANCA | antiTLR4+HMGB1+MPO-ANCA | RAGE Fc+HMGB1  +MPO-ANCA | | anti4+R+HMGB1+MPO-ANCA |
| --- | --- | --- | --- | --- | --- | --- | --- |
| blank |  | .000 | .000 | .001 | .002 | .079 | |
| HMGB1+ANCA | .000 |  | .125 | .003 | .001 | .003 | |
| antiTLR2+HMGB1+MPO-ANCA | .000 | .125 |  | .101 | .041 | .050 | |
| antiTLR4+HMGB1+MPO-ANCA | .001 | .003 | .101 |  | .670 | .412 | |
| RAGE Fc+HMGB1+MPO-ANCA | .002 | .001 | .041 | .670 |  | .602 | |
| anti4+R+HMGB1+MPO-ANCA | .079 | .003 | .050 | .412 | .602 |  | |

**Table S3. Statistical differences between groups in Figure 7**

A

|  | isotype | blank | HMGB1 | MyD88 blocking peptide+HMGB1 | BAY 11-7082+HMGB1 |
| --- | --- | --- | --- | --- | --- |
| isotype |  | .963 | .000 | .022 | .611 |
| blank | .963 |  | .000 | .015 | .561 |
| HMGB1 | .000 | .000 |  | .003 | .000 |
| MyD88 blocking peptide+HMGB1 | .022 | .015 | .003 |  | .062 |
| BAY 11-7082+HMGB1 | .611 | .561 | .000 | .062 |  |

B

|  | blank | HMGB1 | MyD88 blocking peptide+HMGB1 | BAY 11-7082+HMGB1 |
| --- | --- | --- | --- | --- |
| blank |  | .000 | .046 | .122 |
| HMGB1 | .000 |  | .019 | .007 |
| MyD88 blocking peptide+HMGB1 | .046 | .019 |  | .585 |
| BAY 11-7082+HMGB1 | .122 | .007 | .585 |  |

C

|  | blank | HMGB1+PR3-ANCA | MyD88 blocking peptide  +HMGB1+PR3-ANCA | BAY 11-7082  +HMGB1+PR3-ANCA |
| --- | --- | --- | --- | --- |
| blank |  | .001 | .194 | .513 |
| HMGB1+PR3-ANCA | .001 |  | .019 | .005 |
| MyD88 blocking peptide+HMGB1+PR3-ANCA | .194 | .019 |  | .496 |
| BAY 11-7082+HMGB1+PR3-ANCA | .513 | .005 | .496 |  |

D

|  | blank | HMGB1+MPO-ANCA | MyD88 blocking peptide  +HMGB1+MPO-ANCA | BAY 11-7082  +HMGB1+MPO-ANCA |
| --- | --- | --- | --- | --- |
| blank |  | .002 | .180 | .745 |
| HMGB1+MPO-ANCA | .002 |  | .024 | .003 |
| MyD88 blocking peptide+HMGB1+MPO-ANCA | .180 | .024 |  | .296 |
| BAY 11-7082+HMGB1+MPO-ANCA | .745 | .003 | .296 |  |

E

|  | blank | HMGB1+PR3-ANCA | MyD88 blocking peptide  +HMGB1+PR3-ANCA | BAY 11-7082  +HMGB1+PR3-ANCA |
| --- | --- | --- | --- | --- |
| blank |  | .000 | .164 | .707 |
| HMGB1+PR3-ANCA | .000 |  | .020 | .002 |
| MyD88 blocking peptide+HMGB1+PR3-ANCA | .164 | .020 |  | .377 |
| BAY 11-7082+HMGB1+PR3-ANCA | .707 | .002 | .377 |  |

F

|  | blank | HMGB1+MPO-ANCA | MyD88 blocking peptide  +HMGB1+MPO-ANCA | BAY 11-7082  +HMGB1+MPO-ANCA |
| --- | --- | --- | --- | --- |
| blank |  | .000 | .204 | .812 |
| HMGB1+MPO-ANCA | .000 |  | .045 | .002 |
| MyD88 blocking peptide+HMGB1+MPO-ANCA | .204 | .045 |  | .204 |
| BAY 11-7082+HMGB1+MPO-ANCA | .812 | .002 | .204 |  |
