# Supplementary material for: High mobility group box 1 contributes to anti-neutrophil cytoplasmic antibody-induced neutrophils activation through receptor for advanced glycation end products (RAGE) and Toll-like receptor 4
Source: Arthritis Res Ther. 2015 Mar 18;17(1):64. doi: 10.1186/s13075-015-0587-4 (PMC4382936; doi:10.1186/s13075-015-0587-4)
Supplement: Additional file 4: — To confirm the receptors of HMGB1 by gene knockout animals and a neutrophil-like HL-60 cell line transfected with small interfering RNAs. [file 13075_2015_587_MOESM4_ESM.doc]

**Additional file 4**

**To confirm the receptors of HMGB1 by gene knockout animals and a neutrophil-like HL-60 cell line transfected with small interfering RNAs**

**Materials and Methods**

**Reagents**

Recombinant mouse HMGB1 proteins were purchased from eBioscience (San Diego, USA). Recombinant human HMGB1 proteins were purchased from R&D Systems (C23-C45 disulfide C106 thiol form) (Abingdon, UK). The endotoxin level of HMGB1 was below the detection limit (0.125 EU/mL) of the Limulus assay (Sigma-Aldrich, St Louis, USA). PE-conjugated mouse anti-human CD11b, APC-conjugated mouse anti-human CD35, and FITC-conjugated mouse anti-human CD71 were purchased from BioLegend (San Diego, USA), with addition of irrelevant IgG control antibodies. FITC-conjugated mouse anti-human PR3 was purchased from Abcam (Cambridge, UK), with addition of irrelevant IgG control antibody. APC-conjugated rat anti-mouse Ly6G was purchased from BD Biosciences (San Diego, USA). Purified anti-myeloperoxidase (MPO) rabbit polyclonal antibody (ab9535) was purchased from Abcom (Cambridge, UK), reacted with both mouse and human. Fluorochrome dihydrorhodamine was purchased from Sigma-Aldrich (St Louis, USA).

**Mice**

TLR2-/-, TLR4-/-, wild-type C57BL/6 (B6) and C57BL/10 (B10) mice were purchased from the Model Animal Research Center of Nanjing University. The mice were bred and housed in the animal facility at Peking University First Hospital. They were kept under standard conditions of temperature and light, and were fed laboratory chow and water ad libitum.

As previously described by Carlyon et al., murine neutrophils were isolated from 750μL blood from, or wild type mice by centrifugation through an equal volume of Polymorphprep (Nycomed, Oslo, Norway) at 470g for 30 minutes. The neutrophils were removed by aspiration, washed twice with Hanks balanced salt solution without Ca^2+^/Mg^2+^ (HBSS-/-; Beijing, China) and suspended in RPMI 1640 medium, containing 10% fetal bovine serum (FBS) .

**Detection of murine MPO in the supernatant of HMGB1-primed murine neutrophils by ELISA**

Murine MPO in the supernatant of HMGB1-primed murine neutrophils was tested by ELISA using a commercial kit (USCNK, Wuhan, China). Cells were incubated with HMGB1 (10ng/ml) for 30 min at 37°C. The supernatant was collected for ELISA analysis.

**Cell culture and neutrophil-like differentiation**

Human leukemia cell lines HL-60 were obtained from the American Type Culture Collection (ATCC; Rockville, USA). The culture medium used throughout the experiments was RPMI 1640 medium, containing 10% FBS, 2 mM L-glutamine, and 100 U/ml penicillin and streptomycin. Cells were cultured at 37 °C in a humidified chamber containing 5% CO2.

HL-60 cells were differentiated into neutrophils by treatment with 1% DMSO (Sigma-Aldrich, St. Louis, USA). As described previously by Park *et al.*, differentiated HL-60 cells (D-HL-60) were assessed on days 4–7 to determine phenotypic alterations using flow cytometric analysis with a FACScan Accuri C6 (BD, San Diego, USA). D-HL-60 cells were stained with surface antigens, PE-CD11b, APC-CD35, and FITC-CD71. Non-specific staining was assessed with isotype control antibodies .

To assess DMSO-induced granulocytic differentiation of HL-60 cells, we analyzed the expression levels of several surface antigens. On differentiation day 4 (D4), levels of granulocytic differentiation markers CD11b and CD35 were increased to to highest level, while the expression of CD71, a transferring receptor that plays an essential role in cell proliferation, decreased to the lowest level. Based on these results, we chose D4 as an optimum differentiation period and used this time-point for further study.

**Small interfering RNA transfection.**

HL-60 cells, which had been pre-treated with 1% DMSO for 48h, were transiently transfected with small interfering RNA (siRNA) against human TLR2, TLR4, RAGE or scrambled siRNA using Lipofectamine^TM^ RNAiMAX Reagent (Invitrogen, USA) according to the manufacturer's instructions. Briefly, 5μl of Lipofectamine™ RNAiMAX (Invitrogen) and 100 nM siRNA were diluted with Opti-MEM, pre-incubated at room temperature for 20 min and then added to the culture medium. Cells were incubated at 37°C for 48 h with siRNA-lipofectamine complexes and were subsequently harvested for further analysis .

**Measurement of membrane expression of PR3 on D-HL-60 after priming**

Flow cytometry was used to evaluate membrane-bound PR3 expression on D-HL-60. Cells were incubated with HMGB1 or buffer control for 30 min at 37°C. All further steps were performed on ice and washing steps were carried out using HBSS +/+ containing 1% bovine serum albumin (BSA). TruStain fcX (BioLegend, San Diego, USA) was used in all samples prior to the addition of antibodies to block nonspecific antibodies binding. Next, cells were stained with a saturating dose of FITC-PR3 or with an irrelevant IgG1 control antibody for 30 min. Fluorescence intensity of FITC was analyzed using flow cytometry assessment of ANCA-antigen expression. Samples were analyzed using Accuri C6. The level of membrane-bound PR3 expression was calculated as mean fluorescence intensity (MFI) values.

**Measurement of respiratory burst by oxidation of dihydrorhodamine to rhodamine**

For murine neutrophils, the cells were primed with 10ng/ml HMGB1 for 30 min at 37°C and incubated with Anti-MPO IgGs (5μg/ml) for 1 h at 37°C. Then cells were stained with APC-Ly6G and dihydrorhodamine (1μM), which converts to fluorescent rhodamine 123 (Rho 123) when oxidized. The oxidation reaction was stopped on ice, and neutrophil reactive oxygen species production was analyzed by gating on Ly6G cells and measuring the MFI of Rho 123 by FACScan .

For D-HL-60 with various siRNA, the cells were washed twice and primed with 10ng/ml HMGB1 for 30 min at 37°C and incubated with Anti-MPO IgGs (5μg/ml) for 1 h at 37°C. Then cells were stained with dihydrorhodamine (1μM). The oxidation reaction was stopped on ice, and reactive oxygen species production was analyzed by measuring the MFI of Rho 123 by FACScan.

**Statistical analysis**

Quantitative data were expressed as means±SD (for data that were normally distributed) or median and quartiles (for data that were not normally distributed) as appropriated. Differences of quantitative parameters between groups were assessed using one-way ANOVA analysis (for data that were normally distributed) or Mann-Whitney U test (for data that were not normally distributed) as appropriate. Differences were considered significant when P<0.05. Analysis was performed with SPSS statistical software package (version 13.0, Chicago, USA).

**Result**

**Release of MPO by HMGB1-primed murine neutrophils was dependent on TLR4 rather than TLR2**

As shown in Additional file 5: Figure S2, compared with non-primed murine neutrophils, the concentration of MPO was significantly higher in the supernatant of neutrophils which were from B6 or B10 wild type mice, primed with HMGB1 at concentration of 10ng/ml (15038.65± 4723.09pg/ml vs. 38512.21±17029.14pg/ml, *P*=0.009; 10047.66±5507.06pg/ml vs. 17364.29±8135.69pg/ml, *P*=0.012, respectively). There was no significant difference in the concentration of MPO in the supernatant of the neutrophils from TLR4-/- mice between non-primed murine neutrophils and HMGB1-primed neutrophils (7991.74±4369.40pg/ml vs. 5313.03±3821.73pg/ml, *P*=0.332), while the concentration of MPO in the supernatant of HMGB1 primed-neutrophils from TLR2-/- mice was significantly higher than that of non-primed neutrophils (52153.25±14255.794pg/ml vs. 24744.58±7687.02pg/ml, P=0.002).

**Anti-MPO IgGs-induced respiratory burst in HMGB1-primed murine neutrophils was dependent on TLR4 rather than TLR2**

We further studied respiratory burst induced by Anti-MPO IgGs of HMGB1-primed murine neutrophils (Ly6G+ cells). Compared with non-primed neutrophils, the MFI of Rho 123 (representing the amount of generated reactive oxygen radicals) increased significantly in HMGB1-primed neutrophils, which were from B6 or B10 wild type mice, further induced by Anti-MPO IgGs. No obvious respiratory burst activity was observed in cells treated with HMGB1 or Anti-MPO IgGs alone.

Compared with non-primed neutrophils, the MFI of Rho 123 of the neutrophils from TLR4-/- mice, stimulated by HMGB1 plus Anti-MPO IgGs had no significant difference (2749.92±315.54 vs. 2805.17±488.85, *P*=0.848) (**Additional file 6: Figure S3F**), while the MFI of the neutrophils from TLR2-/- mice, stimulated by HMGB1 plus Anti-MPO IgGs, was significantly higher (2667.42±261.75 vs. 4311.67±371.40, *P*<0.001) (**Additional file 6: Figure S3C**).

**Expression of membrane-bound PR3 on HMGB1-primed neutrophils-like HL-60 cells was dependent on TLR4 and RAGE rather than TLR2**

As shown in Additional file 8: Figure S5A, compared with non-primed D-HL-60 cells transfected with control RNA, the level of membrane-bound PR3 expression was significantly higher in these cells primed with HMGB1 at concentration of 10ng/ml (3341.25±399.66 vs. 5187.25±1130.28, *P*=0.005). Compared with HMGB1-primed D-HL-60 cells transfected with control RNA, the levels of membrane-bound PR3 expression on the HMGB1-primed D-HL-60 transfected with TLR4 siRNA and RAGE siRNA were significant lower (5187.25±1130.28 vs. 3209.50±105.10, *P*=0.026; 5187.25±1130.28 vs. 3383.250±457.27, *P*=0.046, respectively), while the levels of membrane-bound PR3 expression on the HMGB1-primed D-HL-60 transfected with TLR2 siRNA had no difference. (5187.25±1130.28 vs. 4682.00±1320.21, *P*=0.409).

**Anti-MPO IgGs-induced respiratory burst in HMGB1-primed neutrophils-like HL-60 cells was dependent on TLR4 and RAGE rather than TLR2**

Compared with non-primed D-HL-60 cells transfected with control RNA, the MFI of Rho 123 increased significantly in D-HL-60 cells transfected with control RNA stimulated by HMGB1 plus anti-MPO IgGs. No obvious respiratory burst activity was observed in cells treated with HMGB1 or anti-MPO IgGs alone.

Compared with D-HL-60 cells transfected with control RNA stimulated by HMGB1 plus Anti-MPO IgGs, the MFI of Rho 123 of the D-HL-60 transfected with TLR4 siRNA or RAGE siRNA stimulated by HMGB1 plus Anti-MPO IgGs were significant lower (496.33±57.59 vs. 387.33±14.19, *P*=0.003; 496.33±57.59 vs. 388.67±12.06, *P*=0.004, respectively). There was no significant difference in the MFI of Rho 123 between D-HL-60 cells transfected with control RNA stimulated by HMGB1 plus MPO-ANCA-IgGs and D-HL-60 transfected with TLR2 siRNA stimulated by HMGB1 plus MPO-ANCA-IgGs (496.33±57.59 vs. 482.00±23.07, *P*=0.603) (**Additional file 7: Figure S4B**).

**Reference**

1. Carlyon JA, Akkoyunlu M, Xia L, Yago T, Wang T, Cummings RD, McEver RP, Fikrig E: **Murine neutrophils require alpha1,3-fucosylation but not PSGL-1 for productive infection with Anaplasma phagocytophilum**. *Blood* 2003;**102**:3387-3395.

2. Park YS, Lim GW, Cho KA, Woo SY, Shin M, Yoo ES, Chan Ra J, Ryu KH: **Improved viability and activity of neutrophils differentiated from HL-60 cells by co-culture with adipose tissue-derived mesenchymal stem cells**. *Biochem Biophys Res Commun* 2012;**423**:19-25.

3. Kim BM, Choi YJ, Han Y, Yun YS, Hong SH: **N,N-dimethyl phytosphingosine induces caspase-8-dependent cytochrome c release and apoptosis through ROS generation in human leukemia cells**. *Toxicol Appl Pharmacol* 2009;**239**:87-97.

4. Kitagawa J, Hara T, Tsurumi H, Ninomiya S, Ogawa K, Adachi S, Kanemura N, Kasahara S, Shimizu M, Moriwaki H: **Synergistic growth inhibition in HL-60 cells by the combination of acyclic retinoid and vitamin K2**. *J Cancer Res Clin Oncol* 2011;**137**:779-787.

5. Carlsen ED, Hay C, Henard CA, Popov V, Garg NJ, Soong L: **Leishmania amazonensis amastigotes trigger neutrophil activation but resist neutrophil microbicidal mechanisms**. *Infect Immun* 2013;**81**:3966-3974.

**Figure legends**

**Additional file 5: Figure S2**. **Release of MPO by HMGB1-primed murine neutrophils from TLR2-/- and TLR4-/- mice**

HMGB1 increased concentration of MPO in the culture supernatant of neutrophils from TLR2 -/- mice as wild type mice (A).

HMGB1 could not increase concentration of MPO in the culture supernatant of neutrophils from TLR4 -/- mice as wild type mice (B).

Bars represent mean±SD of repeated measurements on neutrophils of 5 independent experiments and mice.

**Additional file 6: Figure S3. Anti-MPO IgGs-induced respiratory burst in HMGB1-primed murine neutrophils from TLR2-/- and TLR4-/- mice**

Murine neutrophil respiratory burst induced by Anti-MPO IgGs was measured by conversion of dihydrorhodamine to Rho-123 in HMGB1-primed cells (C, F).

A-B and D-E were representative Flow cytometry results. The percentage of Ly6G+Rho+ neutrophils were regarded as level of respiratory burst.

Bars represent mean±SD of repeated measurements on neutrophils of 3 independent experiments and mice.

**Additional file 7: Figure S4. Expression of membrane-bound PR3 on HMGB1-primed neutrophils-like HL-60 cells and Anti-MPO IgGs-induced respiratory burst in HMGB1-primed neutrophils-like HL-60 cells transfected with TLR2, TLR4 or RAGE siRNA**

HMGB1 increased expression of membrane-bound PR3 on neutrophils-like HL-60 cells transfected with TLR2, TLR4 or RAGE siRNA (A).

Anti-MPO IgGs-induced respiratory burst in HMGB1-primed neutrophils-like HL-60 cells transfected with TLR2, TLR4 or RAGE siRNA (B).

Bars represent mean±SD of repeated measurements on neutrophils of 3 independent experiments.
